# B cell responses to CD40 stimulation ignite anti-tumor immunity by activating CD8^+^ T cell cytotoxicity

**DOI:** 10.1016/j.celrep.2026.117957

**Published:** 2026-09-11

**Authors:** Kendrick Nguyen, Monika Quackenbush, Sara Basbous, Haley Rodriguez, Georgia Lattanzi, Elsie Quansah, Chynna Bowman, Sammy Weiser Novak, Daniela Boassa, Jingting Yu, Taylor Ozbun, Asona Lui, Aatish Thennavan, Peter Watson, April Wehner, Daniel Hollern

**Affiliations:** 1 Nomis Center for Immunobiology and Microbial Pathogenesis, Salk Cancer Center, Salk Institute for Biological Studies, La Jolla, CA 92037, USA; 2 University of California, San Diego, School of Biological Sciences, San Diego, CA 92093, USA; 3 Department of Systems Biology, UT MD Anderson Cancer Center, Houston, TX 77030, USA; 4 BC Cancer Deeley Research Centre, Victoria, BC V8R 6V5, Canada; 5 University of California, San Diego, Department of Radiation Medicine & Applied Sciences, San Diego, CA 92093, USA; 6 Waitt Advanced Biophotonics Center Core Facility, Salk Institute for Biological Studies, La Jolla, CA 92037, USA; 7 Senior author; 8 These authors contributed equally; 9 Lead contact

## Abstract

B cell responses to cancer are critical to patient survival and response to therapy. Yet, the knowledge as to how B cells can elicit anti-tumor immunity remains incomplete. We identify that CD40 pathway activation in B cells in the presence of high tumor mutation burdens activates an anti-tumor B cell response. Significantly, our study shows that CD40-stimulated B cells drive tumor infiltration, organization, and activation of cytotoxic function in CD8^+^ T cells. These results provide key advances in the mechanistic regulation and function of B cell and CD8^+^ T cell responses to cancer.

## In brief

CD40-stimulated B cells drive infiltration, organization, and cytotoxic function of CD8^+^ T cells in triple-negative breast tumors, advancing our understanding of how B cell interactions with CD8^+^ T cells can influence anti-tumor responses.

## Graphical Abstract

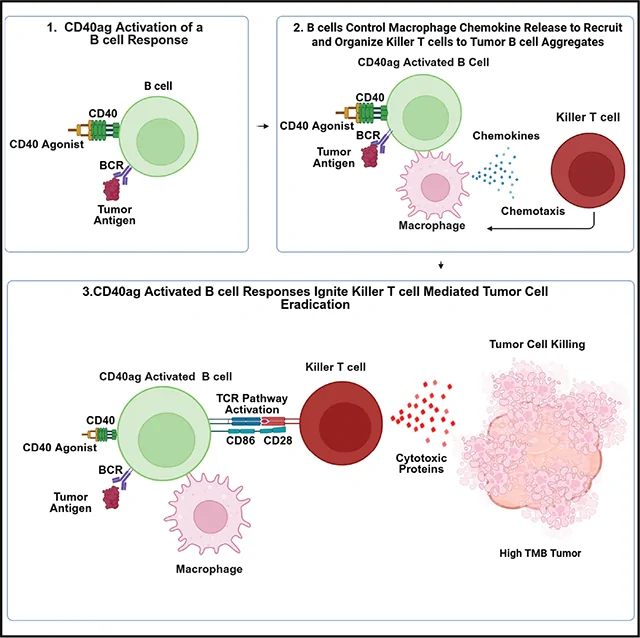

## INTRODUCTION

Standard-of-care cancer treatments are focused on direct targeting of T cell exhaustion checkpoints^1,2^ to restore helper CD4^+^ T cell immune responses^3–5^ and to promote CD8^+^ T cell cytotoxicity.^6,7^ Demonstrating the capacity of the immune system to treat cancer, T cell checkpoint inhibitors have provided incredible benefit to patients with certain types of solid tumor malignancies.^8–10^ However, despite the immense impact of T cell checkpoint inhibitors, a significant proportion of patients fail to gain benefit from these treatments.^11^ This emphasizes the urgent need to identify new mechanisms to enforce anti-tumor T cell responses in patients.

B cells harbor powerful anti-tumor capacity and potential to enhance conventional and novel therapies.^12–17^ However, the regulation and function of B cells in T cell-driven anti-tumor immunity have not been fully elucidated. Because of this gap in research, clinical exploitation of B cell responses for reliable tumor eradication in patients has been limited.

Emphasizing the importance of B cells in cancer biology, studies show that B cells are critical predictors of patient survival and response to therapy.^18–30^ B cell diversity is often shaped in the lymph nodes by germinal center (GC), extrafollicular, or other GC-independent pathways.^31–35^ In lymph nodes, GCs occur in secondary follicles. In tumors, GCs are often found in tertiary lymphoid structures (TLS), which are frequently predictive of favorable clinical outcomes.^18,22–29,36–40^ In part, TLSs may have a favorable prognosis due to the ability of GCs to select B cells with B cell receptors optimized for strong antigen affinity^31,41,42^; likely producing memory B cells and antibody-secreting B cells (plasmablasts, plasma cells) with improved tumor recognition capacity.^43,44^ Prior to and often distinct from GCs,^32^ extrafollicular B cell responses occur with Ki67 + B cells in the medullary zones^32,33^ and the subcapsular sinus^45^ of lymph nodes. Extrafollicular responses are well recognized to produce plasmablasts from memory B cell precursors.^32–34^ There is also a GC-independent B cell response pathway where memory B cells arise prior to GCs from activated B cell precursors at the T/B border in lymph nodes.^35^ Although recent studies indicate memory B cells are important to patient survival and response to therapy,^46–48^ the regulation and function of memory B cell responses in cancer have not been fully elucidated.

Tumor antigen and T cell interactions are key mediators of anti-tumor B cell potential.^13,14,26,49^ A critical T cell signal to B cells is CD40 ligand (CD40LG), which activates the CD40 pathway in B cells. This pathway can shape the balance and timing of GC and non-GC B cell responses.^32,35,42,50,51^ The formation of mature B cell subsets such as regulatory B cells, plasma cells, and memory B cells, can also be affected.^49,50,52–54^ Yet, how CD40 pathway activation shapes the anti-tumor B cell response has mainly been examined in terms of CD40 agonism’s effect on B regulatory cells^55^ and vaccine strategies.^56^

Understanding the function of CD40 activation in B cells is critical given that CD40 agonists (CD40ag), which mimic CD40LG, are active in clinical trials, including triple-negative breast cancer (TNBC).^57–60^ In response to this clinical importance and current gaps, we leveraged a CD40ag antibody to study CD40 pathway activation of B cells. To account for tumor heterogeneity, including the possible role of antigens in these responses, we used multiple low- and high-tumor mutation burden (TMB, which produces neoantigens^61–63^) mouse models that have similar genomics and transcriptomics as human TNBC.^13,64–66^ We determined that anti-tumor B cell responses can occur in high TMB TNBC models with CD40 activation (via CD40ag). We found that this B cell response expresses markers of prognostically relevant atypical memory B cells and can function to mobilize, organize, and activate CD8^+^ T cell responses. Together, our results deliver significant advances to the paradigms describing the regulation and function of B cells and anti-tumor CD8^+^ T cell responses in cancer.

## RESULTS

### CD40 and CD40 ligand are increased in human and murine triple negative breast cancer

In our prior study, we found that Cd40lg was a part of a B cell-T cell interaction signature that predicts responses to chemotherapy and immune therapy.^13^ This prompted further investigation of the significance of this pathway in breast cancer and anti-tumor immunity. In breast cancer, analysis of TCGA mRNA-seq data indicates that basal-like TNBCs have significantly higher expression of CD40LG and CD40 mRNA compared to other breast cancer subtypes (Figures S1A and S1B). CD40 mRNA was linked to increased overall and relapse-free survival across breast cancer subtypes and in TNBC (Figure S1C). In pre-treatment tumors from human TNBC clinical trials,^67–69^ increased expression of CD40 as well as CD40LG was associated with a favorable response to neoadjuvant chemotherapy as indicated by residual cancer burden (RCB) and pathological complete response (pCR) (Figure 1A, supplemental statistics contain analysis of additional human breast cancer datasets). Together, these results suggest that CD40 pathway activity is high in TNBC and potentially relevant to clinical outcomes.

Investigating the CD40 pathway in TNBC mouse models, we observed that Cd40 and Cd40lg mRNAs were elevated in tumors with high TMB compared to low TMB (Figure 1B).^13^ This suggested the importance of high TMB/neoantigen in possibly promoting the activity of Cd40lg^+^ and Cd40^+^ immune cells. To investigate which Cd40^+^ and Cd40lg^+^ cells were active in these high TMB mouse models of TNBC, we performed single-cell sequencing (scRNA-seq) on primary breast tumors. Here, Cd40lg was expressed by CD4^+^ T cells (Figure 1C), and Cd40 was expressed by B cells, macrophages, and dendritic cells (Figure 1C), as described in prior studies.^71–76^ These results, together with studies showing that CD40 provides an activating signal to these immune cells,^77–79^ led us to hypothesize that activating the CD40 pathway in the presence of high TMB/neoantigen would elicit an anti-tumor response.

### CD40 pathway activation drives anti-tumor immunity in high tumor mutation burden TNBC cell lines

To determine whether CD40 activation induces an anti-tumor immune response, we orthotopically implanted TNBC models in mice and used CD40ag antibodies (Rat IgG2A FGK4.5 clone) to provide a molecular mimic of the CD40LG signal with relevance to CD40ag therapeutics.^57^ We systemically administered CD40ag once tumors reached a diameter of 5 mm (Figure S1D). Using KPB25Luv (p53/Brca1-null, Basal-like, high TMB) along with T11-Apobec (p53-null, Claudin-low, High TMB) TNBC tumor cell lines,^13,64–66^ we found that CD40ag significantly improves overall survival in tumor-bearing mice relative to non-treated and Rat IgG2A isotype controls (Figure 1D; Figure S1D). In TNBC tumor transplant models 2224L (p53-null, basal-like, Low TMB) and 2225L (p53-null, basal-like, Low TMB),^13,65,66^ CD40ag provided no survival benefit (Figure 1D). These results suggest that the presence of high TMB (i.e., tumor neoantigens) could be a critical component of CD40 and CD40LG (CD40ag) signaling function in anti-tumor immune responses.

CD40LG and CD40ag can signal to multiple CD40^+^ immune cells, including dendritic cells, macrophages, and B cells.^80^ Moreover, prior studies have demonstrated that CD40 agonism can produce different types of immune responses, such as T cell-dependent or independent anti-tumor immunity in mouse cancer models.^32,34,55,71,75,81,82^ Thus, we used scRNA-seq and bulk RNA-seq of tumors responding to systemic CD40ag administration to obtain an agnostic and comprehensive measurement of the acute anti-tumor response after 9 days following three doses of CD40ag (Figure 1E; Figures S1E and S1F). In the KPB25Luv TNBC model, scRNA-seq indicated that CD40 agonism invoked robust B and T cell tumor infiltration, a dramatic reduction in tumor cells, and altered the frequency of macrophages and dendritic cells in the tumor (Figure 1E; Figure S1F). Bulk RNA-seq of KPB25Luv tumor-draining lymph nodes (TDLNs) also showed CD40ag-driven elevation of Cd40 mRNA and markers for B cells, macrophages, and dendritic cells. (Figure S1E). Similar results were obtained by bulk RNA-seq of T11-Apobec tumors (Figure S1E). Thus, multi-omics analysis indicated that systemic CD40ag can greatly expand B cells while also impacting macrophages and dendritic cells in mouse high TMB TNBCs and TDLNs.

### CD40 pathway activation elicits an anti-tumor B cell response

Given the 3-fold increase in B cell infiltration into the TME following CD40 activation (Figure 1E), we sought to evaluate the role of B cells in the CD40ag-responsive TNBC models. To do this, we inactivated B cells using anti-CD19 (Figure S2A ^13^) or depleted B cells with anti-CD20^13^ monoclonal antibodies. First, blocking the B cell response using anti-CD19 or anti-CD20 in KPB25Luv reduced mouse tumor survival relative to non-treated and isotype controls, independent of CD40ag treatment (Figure S2B). This indicated existing anti-tumor B cell potential in this model. To determine if CD40 agonism enhances anti-tumor immunity by leveraging B cell function, we evaluated the impact of B cell inhibition on CD40ag-driven anti-tumor responses (Figure 2A; Figures S2C and S2D). These tests in KPB25Luv and T11-Apobec revealed that the initially observed anti-tumor effects by CD40ag were lost when B cells were either inactivated or depleted (Figure 2A; Figures S2C and S2D); most notably in KPB25Luv (Figure 2A). Together, these results indicate that there are malignancies where the anti-tumor benefit from CD40ag depends on B cell activity and could be influenced by the presence of high TMB/neoantigen.

To identify the B cell subsets functioning in an anti-tumor role, we immunophenotyped KPB25Luv and T11-Apobec TNBCs after 9 days of CD40ag treatments (3 total doses). By scRNA-seq of KPB25Luv tumors and bulk RNA-seq of KPB25Luv TDLNs, we found that CD40ag induced a dominant Ighm+ B cell response with high expression of activation markers as compared to non-treated tumors (Figure 2B; Figure S2E). Yet, the CD40ag-responsive B cells did not express markers of GC B cells or tumor-infiltrating plasma cells (Figure 2B; Figure S2E, supplemental statistics). CD40-activated B cells in the KPB25Luv tumor displayed significantly elevated markers of memory and atypical memory B cells such as CD180, CCR6, FCRL5, and IL9R^33,47,84–89^ (Figure 2B). Similarly, in the TDLN, flow cytometry and bulk RNA-seq provided evidence for a non-GC B cell response that displays increased expression of memory and atypical memory B cell markers in response to CD40ag (Figures 2C and 2D; Figures S2E–S2H). Similar results were found in the T11-Apobec TNBC model by flow cytometry of B cells from TDLNs (Figure S2I).

Given the expression of genes and proteins typically associated with memory-like and atypical memory-like B cells, we used gene set enrichment analysis (GSEA)^83^ on KPB25Luv tumor scRNA-seq results to evaluate whether these B cells were significantly enriched for features of established gene signatures of memory B cells. GSEA demonstrated significant enrichment for gene expression signatures that are conserved between mouse and human memory B cells^84,85^ (Figure 2E). CD40ag-responding B cells were also enriched for signatures of FCRL5+ B cells and CCR6+ B cells, including a signature for human tumor-infiltrating atypical memory B cells that are prognostically favorable across human cancer types (Figure 2E).^46^

To evaluate whether the markers of the CD40ag-driven B cell responses can predict patient outcomes, we tested the top 100 differentially expressed genes of the CD40ag-activated B cells in multiple TNBC clinical trial datasets (Figures S2J and S2K; supplemental statistics).^67–69^ Our analysis showed that the markers of B cells responding to CD40 agonism consistently predicted beneficial patient responses.

To assess the structural organization of the observed non-GC B cell response, we performed confocal imaging on tumors and TDLNs 9 days after CD40ag treatment (3 total doses). In time-matched, non-treated and isotype controls, immunofluorescent (IF) staining of KPB25Luv TDLNs revealed secondary follicles with Ki67 staining and cellular organization indicative of GCs (Figure 2F; Figure S2L). Despite the GC activity in non-treated and isotype-treated TDLNs, there were few tumor-infiltrating B cells in comparison to CD40ag (Figures 2F and 2G; Figures S2L and S2M). In contrast, KPB25Luv TDLNs lacked secondary follicles and GC organization after CD40ag treatments (Figure 2F). Additionally, there were B cells outside the follicle that lacked Ki67 positivity (Figure S2N). In the tumor, CD40ag induced robust B cell aggregates that lacked GC organization and had higher Ki67 staining relative to non-treated and isotype controls (Figures 2F and 2G; Figures S2L and S2M). Together, these results suggest that CD40ag induces a non-GC anti-tumor B cell response.

### CD40 agonist-driven B cell responses promote a pro-inflammatory tumor microenvironment

To reveal the components of the anti-tumor immune response that were dependent on the B cell response elicited by CD40 activation, we performed scRNA-seq of KPB25Luv tumors with CD40ag + anti-CD19 B cell inhibition (9 days post treatment, Figure 3A). Notably, the addition of anti-CD19 to CD40ag resulted in loss of tumor-infiltrating B cells and many components of the immune response (Figures 3A and 3B). The striking T cell response driven by CD40ag was significantly altered when anti-CD19 was combined with CD40ag (Figure 3B; Figures S3A and S3B); particularly evident was the reduction of tumor-infiltrating T cells from the stem-like CD8^+^ T cell cluster (Figure 3B). Indeed, flow cytometry (Figure 3C) and multi-color immune-fluorescence (Figures S3C and S3D) demonstrated that the CD40ag enhancement of the CD8^+^ T cell response in the tumor was lost following anti-CD19 treatment (relative to non-treated in Figure 3C and relative to isotype control-treated mice in Figures S3C and S3D). Adding anti-CD19 to CD40ag also altered tumor-infiltrating CD4^+^ T cell (Figures 3B and 3D), stem-like CD8^+^ T cell (Figures 3B and 3E), and cytotoxic CD8^+^ T cell gene expression (Figures 3B and 3F). In stem-like CD8^+^ T cell clusters, CD40ag induced genes associated with T cell survival^90–95^ in comparison to non-treated and CD40ag/anti-CD19 tumors (Figure 3E). In the cytotoxic CD8^+^ T cell cluster, CD40 activation elevated *Prf1* (perforin, a gene essential to tumor cell killing^96^) in comparison to non-treated and CD40ag/anti-CD19 treated tumors (Figures 3B and 3F). In addition, we noted that anti-CD19, in addition to CD40ag, led to elevation of exhaustion markers in the tumor-infiltrating cytotoxic CD8^+^ T cell cluster (Figure 3F).

To predict the functional importance of the CD40ag and B cell-driven T cell response, we used the top 100 markers of these CD40ag-induced stem-like and cytotoxic CD8^+^ T cells as a gene signature. We found that the signatures exhibited a significant link to TMB in mouse models (Figure S3E) and response to neoadjuvant chemotherapy in patients with breast cancer (Figure S3F, supplemental statistics). Thus, we next sought to test the anti-tumor potential of these B cell-influenced T cell responses in the KPB25Luv TNBC mouse model.

By depleting CD4^+^ T cells, we observed an initial survival benefit, which was lost over time, leading to a non-significant impact on CD40ag-elicited tumor control (Figure 3G); likely due to CD40ag replacing the CD40LG function of CD4^+^ T cells. In contrast, the anti-tumor effect of CD40ag was dependent on CD8^+^ T cells (Figure 3G). Given that anti-CD19 combined with CD40ag significantly reduced CD8^+^ T cell and B cell infiltration in the tumor (Figures 3A–3C; Figure S3C and S3D), we tested if systemic CD40ag anti-tumor effects relied on lymphocyte egress from lymph nodes using an S1P1R inhibitor FTY720.^75^ FTY720 completely abolished CD40ag-mediated tumor control (Figure S3G); highlighting the importance of B and T cell responses originating from the TDLN to systemic CD40 agonism anti-tumor effects. Given these results and the B cell-reliant CD8^+^ T cell response, we sought to investigate the extent of B cell and CD8^+^ T cell interactions in tumors and TDLNs.

To determine if B cells and CD8^+^ T cells were interacting, we first performed IF staining for CD20 (B cells) and CD8a (CD8^+^ T cells) on KPB25Luv tumors along with TDLNs after 9 days of CD40ag treatment. In the LN follicle, B cell-CD8^+^ T cell colocalization was detected in age-matched tumor-free mice (Figure S4A), non-treated tumor-bearing mice (Figure S4B), isotype control treated tumor bearing mice (Figures 4A and 4B), and CD40ag-treated tumor-bearing mice (Figures 4A and 4B). However, isotype control (Figures 4A and 4C) and non-treated control (Figure S4B) tumors lacked CD8^+^ T cell and B cell colocalization as compared to CD40ag-treated tumors (Figures 4A and 4C). Quantification of the distance between cells demonstrated that B cells and CD8^+^ T cells were in significantly closer proximity in CD40ag-treated tumors relative to isotype controls (Figure 4C). Imaging of CD40ag-treated KPB25Luv TDLNs and tumors with high-resolution confocal microscopy detected CD20 and CD8 signal overlap, suggesting physical contact between CD20^+^ B cells and CD8^+^ T cells (Figure 4D; Figure S4C). To corroborate physical contact of cellular membranes as suggested by confocal microscopy, we used electron microscopy on a region of interest where B cell and CD8^+^ T cell signals overlap by immunofluorescence. Here, electron microscopy of KPB25Luv TDLNs detected B cell-CD8^+^ T cell membrane contact (Figure S4C). We also found evidence of B cell-CD8^+^ T cell aggregates that have CD20 and CD8 signal overlap in human TDLNs and TNBCs (Figure 4E); suggesting that physical contact between B cells and CD8^+^ T cells also occurs in human tumors (Figure 4E). Consistent with these findings, gene signatures of mouse CD40ag-activated B cell and CD8^+^ T cell responses to CD40ag showed significant correlation of single-sample GSEA (ssGSEA) enrichment scores^83,97^ across multiple human breast cancer RNA-seq datasets (Figure S4D, see supplemental statistics for tests in additional datasets).^67–69^ Thus, our results suggest that B cells and CD8^+^ T cells physically interact in mouse and human TNBCs.

### CD40 agonist driven B cell responses cooperate with macrophages to organize CD8^+^ T cells

Given the evidence that B cells and CD8^+^ T cells gain contact with each other, we sought to determine how CD40ag-activated B cells and CD8^+^ T cells establish interaction. ScRNA-seq showed that CD40ag induced macrophages to express elevated Cxcl9 and Cxcl10 mRNAs, which are known to elicit T cell chemotaxis and tumor infiltration (Figure 5A; Figure S5A).^98–102^ Importantly, macrophage Cxcl9 and Cxcl10 mRNAs were significantly lower upon adding anti-CD19 to CD40ag (Figure 5A). In addition, CellChat^103^ predicted that CXCL signaling from macrophages to cytotoxic CD8^+^ T cells is enhanced with CD40ag treatment but is lost when B cells are inactivated (Figure 5B). Moreover, bulk RNA-seq analysis showed significant correlation between gene signatures of these CD8^+^ T cells and pro-inflammatory-like macrophages in breast cancer mouse models (Figure 5C). Thus, we next investigated if the B cell response to CD40 activation could influence macrophage phenotype and function.

To investigate if CD40ag-activated B cells could influence macrophage phenotypes and chemokine secretion, we utilized an *ex vivo* coculture system with B cells isolated from TDLNs of tumor-bearing mice along with macrophages differentiated from bone marrow (BMDMs) of healthy wild-type mice (Figure S5B). Following 9 days of treatment, CD40ag-treated B cells significantly reduced the expression of the M2-like/immune-suppressive-like macrophage marker CD206 in co-cultured macrophages relative to time-matched isotype controls (Figure S5C).^104–106^ Furthermore, analysis of the coculture supernatant by ELISA revealed that CD40-activated B cells can significantly induce macrophage CXCL9 relative to isotype controls (Figure 5D). Notably, CD40ag treatment of BMDMs directly (without CD40ag-treated B cells) was unable to induce CXCL9 production. Together, these results suggest that the CD40ag-activated B cell response can induce a pro-inflammatory macrophage phenotype.

Given that the CD40-activated B cells elicited a chemokine response in macrophages^101,107–109^ (Figures 5A and 5D), we next tested if B cell co-culture with macrophages can induce CD8^+^ T cell chemotaxis using an *ex vivo* Boyden chamber assay. The conditioned media collected from macrophages co-cultured with 9-day CD40Ag-treated B cells induced significant CD8^+^ T cell chemotaxis relative to time-matched isotype control-treated B cells (Figure 5E). Notably, the control CD40ag-treated BMDMs were unable to induce CD8^+^ T cell migration on their own. These results suggest that B cell interactions with macrophages can elicit CD8^+^ T cell chemotaxis.

The ability of CD40-activated B cells to elicit macrophage-mediated CD8^+^ T cell chemotaxis led us to hypothesize that CD40 activation of B cells plays a significant role in CD8^+^ T cell localization to B cell aggregates. By using a multiplex immunofluorescence approach, we stained tumors for F4/80 (macrophages), CD20 (B cells), and CD8a (CD8^+^ T cells) following 9 days of CD40ag. In time-matched isotypes, and aCD19/CD40ag treated tumors macrophages were as prevalent as in CD40ag-treated tumors (Figure 5F; Figure S5D), but B cells, macrophages, along with CD8^+^ T cells, did not appear to organize together as observed in CD40ag-treated tumors (Figures 5F–5H). This could also be seen in non-treated tumors; B cells, macrophages, and CD8^+^ T cells also did not organize together despite an apparent abundance of these cells excluded to the tumor stromal margins (Figure S5E). In stark contrast to isotype and non-treated controls, CD40ag-treated tumors had dense CD20^+^ (B cells), F480^+^ CXCL9^+^ (macrophages), CD8^+^ (T cells) aggregates that were intratumoral (Figure 5F; Figure S5E). CD40ag resulted in shorter distances between macrophages and CD8^+^ T cells compared to isotype, but not aCD19/CD40ag (Figure 5G). In addition to this, CD40ag elicited shorter distances of CD8^+^ T cells (Figure 5H) relative to the isotype control and anti-CD19/CD40ag groups. Indeed, showing a role for B cell activation in organizing CD8^+^ T cells into aggregates, inhibiting B cell responses to CD40ag with anti-CD19 resulted in loss of visible CD20^+^, F480^+^ CXCL9^+^, CD8^+^ aggregates (Figure 5F; Figure S5E). Together, our results suggest that the CD40-activated B cell response coordinates with pro-inflammatory macrophages to recruit and organize CD8^+^ T cells to B cell aggregates in the tumor.

### CD40 agonist driven B cells activate CD8^+^ T cell cytotoxic function

The interaction we have observed between B cells and CD8^+^ T Cells (Figure 4D; Figure S4C) led us to hypothesize that B cells could directly signal to activate CD8^+^ T cells. To predict the mechanism, we leveraged CellChat analysis of scRNA-seq between tumoral B cells and CD8^+^ T cells. Results from this analysis suggested that CD40ag-activated B cells may stimulate CD8^+^ T cell T cell receptors (TCRs) and provide co-stimulatory signals such as CD86^110^ to CD8^+^ T cells (Figure 6A; Figure S6A). Thus, we sought to verify the extent to which CD40-activated B cells could activate CD8^+^ T cells.

To identify if CD8^+^ T cells were activated when they are colocalized with B cells, we performed confocal imaging of tumor-infiltrating B cells and CD8^+^ T cells to evaluate phosphorylation of ZAP70 (pZAP70); noting that ZAP70 is a member of the TCR signaling complex, which is phosphorylated upon TCR activation.^112,113^After 9 days of treatment, we noted that isotype controls contained very few tumor-infiltrating B cells colocalized with CD8^+^ T cells (Figure 6B). In time-matched CD40ag-treated samples, tumor-infiltrating B cells colocalized with CD8^+^ T cells in aggregates, and phosphorylated ZAP70 could be observed on CD8^+^ T cells (Figure 6B; Figure S6B). B cells within these aggregates also had phosphorylated ZAP70 (Figure 6B); noting that ZAP70 has been seen in prior studies to be expressed in activated B cells.^113–116^ To evaluate if CD40 agonist-treated B cells can directly induce TCR activation, we used an *ex vivo* co-culture of isotype control or CD40-activated B cells isolated from KPB25Luv TDLNs with tumor naive CD8^+^ T cells and measured phosphorylated ZAP70. Here, CD40ag-treated (9 days) B cells induced significant phosphorylation of ZAP70 in CD8^+^ T cells compared to isotype controls as measured by immunofluorescence MFI (Figures 6C and 6D). Blocking CD8a (a TCR signaling component^117^) abrogated CD40ag-treated B cells’ ability to activate CD8^+^ T cell phosphorylation of ZAP70 (Figure 6D); adding further evidence that B cells are stimulating CD8^+^ T cell TCRs. These findings prompted us to investigate whether CD40ag-treated B cells could subsequently influence CD8^+^ T cell cytotoxic function.

To interrogate the functions of CD8^+^ T cells that had been activated by CD40-stimulated B cell responses, we leveraged a co-culture system where we utilized anti-CD3 antibody stimulation of naive CD8^+^ T Cells. As scRNA-seq predicted that CD40ag-treated B cells regulate effector molecule expression in CD8^+^ T cells (Figure 3F), we tested if CD40ag-treated B cells from KPB25Luv TDLNs could influence CD8^+^ T cell secretion of perforin and granzyme B. B cells from CD40ag-treated TDLNs induced significant production of perforin in CD8^+^ T cells relative to non-treated B cells (Figure 6E). CD40ag-activated B cells did induce an increase of granzyme B relative to non-treated B cells, although it was non-significant by ANOVA testing across the four groups (Figure 6E). In agreement with CellChat predictions (Figure 6A), the increase in perforin required B cell co-stimulation through CD86 (Figure 6E). These results suggested that CD40-activated B cells can induce cytotoxic phenotypes in CD8^+^ T cells, leading us to determine if B cells could enhance tumor cell killing function in CD8^+^ T cells.

Given that CD40ag-activated B cell responses were able to signal, activate, and regulate CD8^+^ T cell expression of cytotoxic molecules, we next compared isotype control and CD40ag-treated B cells from KPB25Luv TDLNs for their capacity to induce CD8^+^ T cell-mediated killing of cancer cells (after 9 days of treatment). As one approach, we conducted live cell imaging of our *ex vivo* coculture to monitor CD8^+^ T cell-mediated tumor cell killing (Videos S1 and S2). This showed that CD8^+^ T cells cultured with CD40ag-treated B cells were able to induce significantly higher tumor cell killing, relative to isotype control-treated B cells (Figure 6F). As another approach, we used an Incucyte assay to measure annexin V staining in KPB25Luv tumor cells induced by CD8^+^ T cells co-cultured with B cells. This demonstrated that CD40ag-treated B cells were able to induce significant tumor cell killing by CD8+ T cells relative to isotype control-treated B cells (Figure 6G). Together, our results suggests that B cell responses to neoantigen and CD40LG/CD40ag can organize CD8^+^ T cells, provide signals to induce cytotoxic functions in CD8^+^ T cells, and enhance tumor cell killing abilities in CD8^+^ T cells.

## DISCUSSION

Collectively, our results advance the canonical models of B cell and CD8^+^ T cell regulation and function. These results also provide translationally relevant insights to CD40ag clinical trials.^57,60,118^ We find that the presence of high TMB (neoantigen) and systemic CD40 activation can potentiate an anti-tumor B cell response that is enriched for markers and gene signatures that are associated with memory B cells and atypical memory B cells. As CD40 stimulation in high TMB TNBC model enhanced anti-tumor B cell activity (Figure 2A; Figures S2C and S2D) and promoted the tumor infiltration of both B cells (Figure 1E; Figures 2F and 2G; Figures S2L and S2M) and CD8^+^ T cells (Figure 1E; Figures 3A–3C; Figures S3C and S3D), these findings suggest that direct activation of B cell pathways could enhance both B cell and CD8^+^ T cell-mediated immune responses in high TMB tumors.

Prior studies outside the context of cancer have reported a tendency for CD40 activation to elicit memory B cell formation.^35,81,119^ Based on the expression of CCR6, FCRL5, and gene signatures associated with memory B cell responses (Figures 2B, 2C, and 2E; Figure S2E), our findings on B cells are reminiscent of the recently reported prognostic CCR6^+^ FCRL5^+^ atypical memory B cells found in human cancers.^46–48^ Thus, these B cell responses may be atypical-like rather than conventional memory B cells. Perhaps related to our findings, prior work has shown that the presence of atypical memory B cells enhances the prognostic strength of CD8^+^ T cells.^120^ Our results build on these studies by providing insights regarding the potential regulation and function of CCR6^+^ FCRL5^+^ atypical memory B cells, especially when memory B cells are not anergic.^48,121^ Note that although the B cell responses we observe are non-GC, this does not mean that these B cell responses are extrafollicular.^122^ Eisenbarth et al. advise that the classification of extrafollicular should be avoided unless proliferation of antigen-specific B cells outside the follicle is detected.^33^ Notably, the B cells we detected outside the follicle in CD40ag-treated TDLNs were not Ki67 positive (Figure S2N).

Given the role of CD40–CD40LG signaling in TLS formation and GC activity,^71,123,124^ it may be surprising that CD40ag led to loss of GCs while promoting a B cell response in the TDLN (Figures 2D–2F; Figures S2E, S2H, and S2I) and in the tumor (Figure 2F). Complementary to our results, Taylor et al. identified memory B cell precursors that mature directly into IgM^+^ or IgG^+^ memory B cells in a GC-independent fashion and in response to strong CD40 stimulation.^35^In Taylor et al., the team found that these CD40-driven memory B cell responses occurred prior to GC formation. Though we have not established if the B cell responses are similar to those studied by Taylor et al., we speculate in our study that CD40ag may reduce GC activity by diverting B cells away from GC fate and promoting their maturation into atypical-like memory B cells.

Although control mice and CD40ag-treated mice did not show a dramatic difference in CD8^+^ T cell colocalization with B cells in the follicle of TDLNs, our *ex vivo* experiments showed that CD40ag-treated TDLN B cells elicit significantly higher CD8^+^ T cell activation capacity and CD8^+^ T cell tumor cell killing potential. This suggests that while B cells and CD8^+^ T cells may colocalize in the TDLN, CD40ag signals enhance the ability of B cells to activate CD8^+^ T cells once in these interactions.

Blocking B cell activation had a dramatic impact on CD40ag-mediated CD8^+^ T cell infiltration into the tumor (Figures 3B and 3C; Figures S3C and S3D); suggesting that B cells are a key facilitator of CD8^+^ T cell migration during CD40ag treatment. In the tumor, our evidence suggests that B cells and macrophages may cooperate to both mediate CD8^+^ T cell infiltration into the tumor and promote cellular aggregate formation where they can exchange molecular signals (Figures 5E–5H). In support of this interpretation, we show that CD40 agonist-activated B cells can have a profound impact on macrophage CXCL9 expression and secretion (Figures 5A–5D) as well as macrophage-mediated CD8^+^ T cell chemotaxis (Figure 5E). Further showing the importance of B cells to this process, *ex vivo* and direct treatment of macrophages with a CD40 agonist was unable to produce these effects. Indeed, our results are complemented by recent studies showing that CXCL9^+^ macrophage responses are important to CD8^+^ T cell homing to the tumor^109^; which strongly suggests the underlying mechanism that explains why B cell responses were required for the formation of CD8^+^ T cell aggregates in the tumor in our study (Figure 5F; Figure S5E).

The CD40ag-activated B cell response had overlapping signals between CD20 and CD8 in the TDLN and primary tumor of the KPB25Luv TNBC mouse model (Figure 4D; Figure S4C); with correlative electron microscopy showing membrane contact (Figure S4C). We also find this same colocalization in patient TDLN and TNBC (Figure 4E). We detected evidence that CD40-activated B cells provide signals to CD8^+^ T cells that require surface contact. Namely, we observe that CD40-activated B cells enhance pZAP70 in CD8^+^ T cells (evidence of TCR activation; Figures 6B–6D) and utilize CD86 to enhance perforin expression in CD8^+^ T cells (Figure 6E). Given the evidence for membrane contact and surface-based signal exchange, these results lead us to speculate that CD40-activated B cells may form immune synapses with CD8^+^ T cells.^125,126^ Moreover, evidence of TCR stimulation also raises the possibility that CD40-activated B cells are capable of antigen presentation to CD8^+^ T cells, as CD40-activated B cells can enhance pZAP70 in CD8^+^ T cells (Figures 6C and 6D). However, with our mouse models on the FVB background, we are not able to use genetic approaches that knock out MHC-I in B cells to verify this.

Although our results show that B cells can function in activating CD8^+^ T cell responses, dendritic cells are commonly attributed to be the canonical driver of CD8^+^ T cell responses to cancer (especially in response to CD40ag) [14, 119, 121]. It is important to note that CD8^+^ T cell responses to CD40ag were lost by B cell inhibition, even while dendritic cells were eligible to be activated by the CD40 agonist. Thus, our findings raise the important question of whether B cells and dendritic cells cooperate [145–147] or function as distinct pathways for CD8^+^ T cell activation. One possibility is that B cells are functioning as organizers and helping dendritic cells come into contact with CD8^+^ T cells to initiate dendritic cell priming of CD8^+^ T cells either in the draining lymph node or tumor. Indeed, our existing data show that B cells are critical for CD8^+^ T cell infiltration and organization in the tumor (Figures 5F–5H; Figure S5E). Thus, it is possible that part of the CD8^+^ T cell activation mechanism involves B cells aiding dendritic cell access to CD8^+^ T cells. Yet, other mouse tumor models will have to be used to assess whether B cells can still activate CD8^+^ T cells when dendritic cells have been ablated.

Our results are also relevant to the recent studies in human cancers (including TNBC), which have detected a significant correlation between B cell responses and CD8^+^ T cell activity.^46,120,127,128^ Indeed, we find that B cells form aggregates and contact with CD8^+^ T cells in human triple-negative tumors and TDLNs (Figure 4E). In addition, mouse models of cancer have detected a correlation between B cell responses to neoantigen and tumor-infiltrating CD8^+^ T cell responses.^13,14^ Our results significantly expand these correlative findings with experimental and functional evidence in mouse models, demonstrating that the CD40ag-activated B cell response can functionally promote macrophages to organize CD8^+^ T cells (Figures 5F–5H; Figure S5E) and then help CD8^+^ T cells to be activated (Figures 6B–6D; Figure S6B; Figures 6F and 6G).

Overall, our results expand the paradigm on B cell regulation, heterogeneity, and function. Our study also contributes to a B cell-centric model of anti-tumor immunity by demonstrating how CD8^+^ T cell responses to cancer are regulated by B cells. Relevant to ongoing and future clinical trials,^57–60^ our results underscore the largely untapped opportunity to leverage B cell stimulatory pathways to enforce CD8^+^ T cell activation and CD8^+^ T cell-mediated anti-tumor immunity. Indeed, our findings add to the growing evidence that B cells can be a potential therapeutic tool to improve anti-tumor immunity.^12,13,16,49,56,129–132^

### Limitations of the study

Due to the scarcity of specimens,^60^ a limitation of our study is that the human TNBCs we studied did not receive CD40ag treatment. Another limitation is that we have not deconvoluted the underlying modes that determine the heterogeneity of CD40ag-driven immune responses; which is beyond the scope of this study. Indeed, CD40ag is known to drive B regulatory cells in certain tumor models.^55^ Moreover, CD40ag can drive T cell-dependent anti-tumor immunity and, at other times, T cell-independent anti-tumor effects.^74,75,123,133,134^ Thus, there are tumor contexts where B cells and T cells will respond differently to CD40ag. Even though we detect robust CD40ag responses in high TMB tumors and a lack of response in low TMB tumor models, we cannot generalize these results to indicate that CD40ag will always lack efficacy in low TMB tumors. For example, there are studies that have seen CD40ag inducing an effective anti-tumor response in cancer models that are likely low TMB.^82^ We also note the limitation of our mouse models to address several key aspects of CD40ag response mechanisms. Namely, because our mouse models are FVB and not on a C57 background, we were unable to perturb dendritic cell function to address the contribution of dendritic cells to our results. In addition, our cancer models are high TMB and thus produce many endogenous polyclonal neoantigens of varying mutant allele frequency.^13^ While this is an advantage to mimic human tumors,^9,26,135–138^ these models are limited in the study of antigen-specific B cells and T cells. This likely dampens the capacity to observe a similar magnitude of strength of CD8^+^ T cell effects as can be observed with clonal model neoantigen systems. We have not investigated if CD40ag stimulation of B cells *in-vitro* could elicit the phenotype observed *in vivo* and how these phenotypes compare to B cells stimulated with LPS or CpG. In addition to this, we did not phenotype B cells post-coculture with CD8^+^ T cells. However, the culture conditions were the same for every experimental condition in the co-culture assay, indicating the effect observed was due to CD40 stimulation of B cells. Despite these limitations, our findings herein represent a major advancement in the understanding of how B cell and CD8^+^ T cell responses are regulated and function in the context of high TMB TNBC tumors and CD40 pathway activation.

## RESOURCE AVAILABILITY

### Lead contact

Request for information or access to resources should be directed to the lead contact, Daniel Hollern (dhollern@salk.edu).

### Materials availability

This study did not generate new unique reagents. Request for materials or information pertaining to this study may be directed to the corresponding author, Daniel Hollern (dhollern@salk.edu).

### Data and code availability

- The datasets generated during this study are available at GEO Datasets GSE276004 (Bulk RNA-seq) and GSE276006 (scRNA-seq). These datasets are publicly available as of the date of publication and can also be found in the key resources table. Please see the key resources table for already published datasets and, for more information, contact Daniel Hollern (dhollern@salk.edu).
- This paper does not report any original code. All computational tools used in this study to analyze data are publicly available and have been previously published. Refer to the key resources table for each computational tool.
- For any additional information that is necessary to reanalyze the data reported, contact Daniel Hollern (dhollern@salk.edu).

## STAR★METHODS

### EXPERIMENTAL MODEL AND STUDY PARTICIPANT DETAILS

#### Animals

All animal work was done in accordance with IACUC guidelines and procedures. All the mice utilized for tumor studies were female and were allowed to mature to 12 weeks of age. FVB and Balb/c mice were obtained from Jackson Laboratories. All Tumor transplants were syngeneic. KPB25Luv, 2224L, and 2225L were orthotopically injected into FVB mice whereas T11-Apobecwas injected in a similar fashion into Balb/c mice. Orthotopic injections were done using 500,000 cells of KPB25Luv, 2224L, 2225L, or 200,000 cells of T11-Apobec in a single cell suspension with Matrigel into the number four mammary gland. Tumor growth was assessed 2–3 times a week using caliper measurements and once a tumor reached 5 mm in diameter, mice were randomized to treatment/control groups. Caliper measurements will continue to take place 2–3 times a week until tumors reach 20 mm, which will then be considered end-stage. Only female mice were used in this study, and as a consequence, sex-associated effects were not evaluated.

#### Tumor cell lines

Tumor cell line models used were KPB25Luv, and T11-Apobec. KPB25Luv cells were cultured in complete Humec media with supplement (Gibco, #12752010). This media was supplemented with 5% FBS (Cytiva, #SH30070.03) and 100 U/mL of Penicillin 100 μg/mL of Streptomycin (HyClone, # SV30010). T11-Apobec cells were cultured in RPMI-1640 (Gibco, #11875–119) media supplemented with 5% FBS, 100 U/mL of Penicillin 100 μg/mL of Streptomycin, and 1 μg/mL puromycin (Gibco, #A1113803). These cell lines were all grown in 10 mm tissue culture dishes till about 70–80% confluent and then used for our assays. Samples were sent to IDEXX BioAnalytics for mycoplasma testing and were confirmed negative.

#### Tumor transplant line

2225L and 2224L mouse mammary tumor lines are transplanted models, maintained by passaging in Balb/c mice.^13,65,66^ To prepare for orthotopic injections of mice into the mammary fat pad, excised tumors were cut into 1 mm pieces using a sterile blade and then placed onto a 70-μm filter. Cell suspension was obtained through mechanical disassociation against the 70-μm filter using a syringe plunger. Cells were then used for downstream assays.

### METHOD DETAILS

#### Antibody regimens

Antibody was delivered intraperitoneally in 100 μL volumes bi-weekly for the following: CD40ag (400 μg, Leinco F1195), aCD4 (500 μg, Leinco, #C2838), aCD8 (500 μg, Leinco, #C2848), anti-CD19 (400 μg, Bioxcel, #BE0150), or Rat IgG2a Isotype (400 μg, Leinco, I-1177). Antibody was delivered intravenously in 100 μL volumes once every 20 days for the following: aCD20 (Ultra-LEAF Purified anti-mouse CD20 (250 μg, Biolegend, #152104), and Rat IgG2b Isotype (250 μg,Leinco, # I-1034).

#### FTY720 experiments

FTY720 (Cayman Chemical, #10006292) was diluted in a vehicle solution (98% PEG400, 2% Tween-80) at a 1 to 10 ratio. Once solution is prepared FTY720 (1 mg/kg) was delivered intraperitoneally in 100 μL volumes daily.

#### Flow cytometry

Mouse tumors were digested and turned into a single cell suspension using Miltenyi’s tumor dissociation kit (Miltenyi, 130–096-730). Mouse TDLNs were placed on a 70-μm filter in ice-cold 1x PBS. Single cell suspension of mouse TDLNs were obtained by mechanical dissociation against the 70-μm filter using a syringe plunger. Single cell suspension was then washed with cold 1x PBS and stained with combinations of Abs purchased from different vendors and listed in key resources table. Non-specific staining was blocked by incubation with mouse FC block αCD16/CD32 (BD Biosciences, #553141) for 30 min. For intracellular cytokine labeling cells were incubated for 3 h at 37°C in RPMI-1640 + 10% FBS with PMA (50 ng/mL, Sigma-Aldrich, #P1585), Ionomycin (1 μg/mL, Sigma-Aldrich, #I0634) and Brefeldin A (10 μg/mL, Sigma-Aldrich, #B6542). Before intracellular staining cells were fixed and permeabilized using eBioscience FOX3P/Transcription Factor Staining Buffer Set (Invitrogen, #00–5523-00). Samples were analyzed with a FACSymphony A3 or LSRII analyzer (BD Biosciences) and the FlowJo software (Version 10.10). Memory-like B cells were gated by being B220^+^, CD19^+^, IgM^+^ and IgD low followed by MFI measurements of FCRL5, CCR6 and IL9R as supported by prior publications.^46,47,85,86,88,89,147,148^

#### Immunofluorescence

For formalin-fixed paraffin embedded (FFPE) mouse tumors and TDLNs, tissues were fixed using zinc-based formalin. Fixed tissues were embedded in paraffin and sectioned at 5 μm thickness. Antigen retrieval was performed using a sodium citrate buffer (10 mM sodium citrate, 0.05% Tween 20, pH 6.0), for 25 min at 100°C. Tissues were blocked for 1 h at room temperature (RT) using 10% goat serum in 1% BSA 1X TBS. Tissues were then incubated with Goat F(ab) anti-mouse IgG H&L (Abcam, # ab6668). Tissues were then incubated with Primary antibody staining was done using CD20 (Abcam, #ab271288), Ki67 (Abcam, #ab279653), and CD8a(Abcam, #ab17147) antibodies overnight at 4°C. Fluorophores for AF488, AF568, and AF647 at a 1:500 dilution were used for secondary antibodies to the respective host of the primary antibody and incubated with the samples for 1 h at RT. Nucleus staining using DAPI (ThermoFisher, #62248) was performed for 5 min. Samples were mounted using Prolong Gold Mounting medium (ThermoFisher, #P36930) and cured for at least 48 h before imaging.

The tumors and TDLNs mounted in OCT embedding compound were sectioned at 5 mm using a cryostat and stored at −80°C. Before proceeding to the immunofluorescence staining, the slides were air-dried for 30 min at room temperature (RT) and rehydrated in PBS for 10 min. After fixation with PFA and permeabilization using Tween 20, the nonspecific binding sites were blocked using 10% goat serum diluted in 1% BSA TBS. primary antibody staining was done using anti-CD20 (Abcam, #ab271288), anti-CXCL9 (Abcam, #ab202961), anti-F4/80 (Abcam, #ab6640) or anti-CD8a (Abcam, #ab17147) antibodies overnight at 4°C was applied to the tissues. The sections were washed and incubated for 45 min with secondary antibody fluorophores for AF488, AF568, and AF647 at a 1:200 dilution to the respective host of the primary antibody along with a combination of the following conjugated antibodies: AF647 rat anti-mouse CD20 (1/50, BD biosciences, BD 568251) or AF488 rat anti-mouse CD8 (1/50, Biolegend, #100726). The sections were stained with DAPI (1/1000, Sigma Aldrich, 62248), washed and mounted using the prolong Gold antifade Mountant (Invitrogen, #P36930).

For imaging and analysis, tissues were imaged using an Axioscanner and Fluoview 3000. Analysis was performed with Qupath.^146^ Imaged tissues were segmented by using a pixel classifier thresholder. Cell detections were segmented in tissue annotations using StarDist.^149^ Training images were generated from each biological replicate across all conditions. An object classifier using an artificial neural network was trained on positive and negative detections per channel. Trained object classifiers were applied to all cells detected within tissue annotations to assign cell classification. Distance between cells of a given classification was calculated in QuPath and statistics were performed on individual cells as independent measures. Cell density was determined as the number of positive cell detections per tissue area.

#### Phospho-Zap-70 (pZAP70) immunofluorescence

For formalin-fixed paraffin embedded (FFPE) mice tumors used for immunofluorescence were fixed using zinc-based formalin. Fixed tissues were embedded in paraffin and sectioned at 5 μm thickness. Antigen retrieval was performed using an IHC Antigen retrieval solution with high pH (Invitrogen, #00–4956-58) for 25 min at 100°C. Tissues were blocked for 1 h at room temperature (RT) using 10% goat serum in 1% BSA 1X TBS. Tissues were then incubated with Goat F(ab) anti-mouse IgG H&L (Abcam, # ab6668).Tissues were then incubated with Primary antibody staining was done using CD20 (Abcam, #ab271288), and pZap70 (Abcam, #ab300410) antibodies overnight at 4°C. Fluorophores for AF488, and AF568 at a 1:500 dilution were used for secondary antibodies to the respective host of the primary antibody and incubated with the samples for 1 h at RT. Tissues were then incubated in Rabbit (DA1E) monoclonal antibody IgG Isotype Control (Cell Signaling Technology, #3900) at 10x the concentration of primary pZap70 antibody used for 1 h. Primary conjugated anti-mouse CD8a Alexa Fluor 647 antibody (Abcam, ab237365) were used to stain the tissue for 1 h. Nucleus staining using DAPI (ThermoFisher, #62248) was performed for 5 min. Samples were mounted using prolong Gold antifade Mountant (Invitrogen, #P36930) and cured for at least 48 h before imaging.

For *ex vivo* coculture pZAP70 staining, B cells and CD8 T cells were co-cultured on poly-D-lysine (P6407, sigma Aldrich) coated CellVis C8–1.5H-N 8 well chambered coverglass slide. Following completion of the coculture, cells were directly fixed with 4%PFA. Permeabilized with tween 20, and then washed and blocked for 1 h at room temperature in PBS containing 5% fetal bovine serum (FBS). Following blocking, cells were incubated for 40 min with the following primary antibodies: FITC anti-mouse CD3 (Biolegend, #100204), Alexa Fluor 647 anti-mouse CD20 (BD, #568251), and rabbit anti-phospho-ZAP70 (Cell Signaling Technology, #2717). After washing, cells were incubated with goat anti-rabbit IgG (H + L) Alexa Fluor 568 (Abcam, #ab175471) for 1 h at room temperature. Cells were subsequently washed, stained with DAPI (1 μg/mL, Sigma Aldrich, 62248) for nuclear visualization and imaged using the Airyscan confocal microscope. MFI was measured with ImageJ [136]. At least 50 nuclei were analyzed per image, and the MFI of pZAP70 was quantified on the original images after ROI definition based on DAPI staining. The average fluorescence intensity of pZAP70 was then calculated for each image. At least 100 CD8^+^ T cells were analyzed per biological replicate. Values were normalized to the average background fluorescence intensity, calculated from the MFI of whole images obtained from samples (*n* = 4) processed without primary anti-pZAP70 antibody staining. The values were normalized by dividing the MFI by the average background fluorescence intensity.

#### Correlative light and electron microscopy

Mice were orthotopically implanted with 500,000 cells of KPB25Luv. Once tumors have reached 5 mm in size mice were treated with CD40ag (400 μg, Leinco #F1195) in 100 μL volumes biweekly via intraperitoneal Injections. After Nine days and three doses of CD40ag Mice were euthanized. Tumor draining lymph nodes were identified by necropsy, dissected, and were drop fixed into freshly made room temperature fixation solution (0.1M sodium cacodylate, 4% PFA) and fixed overnight in the same fixative at 4C. Samples were infiltrated with 30% sucrose in PBS over two days and frozen in OCT in isopentane cooled with liquid nitrogen. Frozen 20 μm sections were collected onto Celltak coated slides and prepared for fluorescence imaging. Slides were air-dried for 30 min at room temperature (RT) and rehydrated in PBS for 10 min. The nonspecific binding sites were blocked using PBS completed with 10% fetal bovine serum and then Primary antibody staining was done using CD20 (Abcam, #ab271288), and CD8a (Abcam, #ab217344) antibodies overnight at 4°C was applied to the tissues. The sections were washed and incubated for 45 min with the secondary antibody AF568 goat anti-rat IgG H&L (1/200, Abcam #ab175476) and AF647 goat anti-rabbit IgG H&L (1/200, Abcam #ab150083). The sections were stained with DAPI (1/1000, Sigma Aldrich, 62248), washed and mounted using a glycerol solution (85%, 15% Milli-Q water). All fluorescence imaging was conducted using a Zeiss LSM880 upright system with Airyscan equipped with 20x/0.8 air and 63x/1.4 oil objectives. A low-resolution map of the section was collected and regions of interest containing T cell/B cell interactions were identified. A series of z-stacks were collected at varying lateral and axial resolutions to facilitate the identification of the cell-cell interactions at progressively higher magnifications. After fluorescent imaging, the coverslip was removed from the slide by immersion in a Coplin jar filled with 1X PBS. The tissue was carefully washed with 1X PBS until all mounting solution was removed and carefully encircled with a PAP pen. The lymph node tissue section was flooded with strong fixative (2.5% glutaraldehyde, 4% paraformaldehyde, 3 mM CaCl2 in 0.1M sodium cacodylate buffer) for 30 min then washed five times in cold 0.1M sodium cacodylate buffer containing 3 mM CaCl2. The tissue section was then stained with 1% osmium/1.5% potassium ferrocyanide in 0.1M cacodylate buffer with 3 mM CaCl2 for 1 h then washed five times with cold distilled water. The sample was then serially dehydrated in ice-cold ethanol, rinsed three times in anhydrous ethanol, then serially infiltrated with the hard formulation of Eponate 12 resin by gently flowing ascending concentrations of resin in ethanol over the tissue sample. A gel capsule was placed around the sample and assembly was polymerized in an oven at 60°C overnight. The following day, the gel capsule was back-filled with more resin and the sample was polymerized again in an oven at 60°C overnight. The samples were then released from the slide by immersion between solutions of liquid nitrogen and hot water.

A rectangle was trimmed around the entire embedded tissue and a series of ultrathin sections were cut from the blockface using a Leica UC7 ultramicrotome equipped with Diatome trimming and Histo diamond knives. A ribbon of approximately 90 serial ultrathin sections 100 nm thick was collected onto a silicon chip as previously described.^150,151^ Using double sided carbon sticky tape, the silicon chip was mounted to an aluminum stub and imaged in a Zeiss Sigma VP scanning electron microscope (SEM) with a Gatan backscattered electron detector. Images were collected using the ATLAS 5 control system (FIBICS). The fluorescent images were imported into ATLAS5 and served to identify the area of interest (ROI) for further SEM imaging at higher resolutions. The ROI was correlated between the high resolution Airyscan data and the SEM imaging within the ribbon of serial ultrathin sections by overlaying DAPI signal with nuclei observed by SEM. High resolution images were collected of the correlated regions of interest with a pixel size of 8 nm and a working distance of 7.3 mm using a 30-μm aperture and an accelerating voltage of 3 kV.

#### Pathology

In addition to molecular analysis from flow cytometry, bulk RNA-seq, and single cell sequencing, extrafollicular and germinal center activity was identified by consensus in pathology analysis of stained sections of tumor sentinel lymph nodes. Germinal center activity in secondary follicles was marked by KI67 activity near the mantle zone in the cortical area. Extrafollicular-like activity was marked by the absence of secondary follicles and GCs and KI67 + B cell localization around the medullary sinus chords and subcapsular sinuses.

#### Co-culture of B cells and CD8^+^ T cells

B cells were isolated by magnetic sorting (Miltenyi, Pan B Cell Isolation Kit II mouse #130–104-443) from TDLNs of CD40 treated, isotype control, or nontreated mice, while splenic naive CD8^+^ T cells were isolated from healthy control mice (Miltenyi CD8a T cell Isolation Kit #130–104-075). Some B cells were incubated 30 min at 37°C with10 μg/mL αCD86 (Bioxcel, # BE0025), and some CD8^+^ T cells were incubated 30 min at 37°C with 10 μg/mL αCD8a (Bioxcel, #BE0223). B cells and CD8^+^ T cells were then co-cultured at a 1:2 ratio. Cells were co-cultured in RPMI-1640 (Gibco, #11875–119) supplemented with 10% FBS (Cytiva, #SH30070.03), 100 U/mL of Penicillin 100 μg/mL of Streptomycin (HyClone,# SV30010), 1X NeAA (Gibco, #11140050), 25 mM HEPES (Gibco, #15630080), 1 mM NaPyruvate (Gibco, #11360070), 100 ng/mL IL4 (Biolegend,#574306), 2 μg/mL BAFF (Acro Biosytems,#BAF-M521Y), 0.05 mM 2-Mercaptoethanol (Gibco, #21985023) freshly added to the medium, 10 ng/mL IL2 (Peprotech, #212–12) and 10 ng/mL IL7 (Peprotech, #217–17) were also added to the Media described. Co-culture lasted for 48–72 h. Cells were then analyzed by flow cytometry or immunofluorescence.

#### Live cell imaging CD8^+^ T cell tumor cell killing assay

B cells were isolated from TDLNs of mammary tumor bearing mice using Miltenyi B Cell magnetic isolation kit (Miltenyi, #130–104-443). CD8^+^ T cells were isolated from spleens of FVB mice using a Miltenyi CD8^+^ T cell magnetic isolation kit (Miltenyi, #130–104-075) and stimulated using an anti-CD3 antibody (BD, # 553057). B cells and CD8 T cells were co-cultured at a 1:2 ratio for 48 h. The next day 40.000 tumor cells were seeded in 8 well plate pre-coated with poly-L-Lysine (ibidi, #80804) and allowed to adhere overnight. To assess the cytotoxicity potential of CD8^+^ T cells derived from these B-T cocultures we isolated CD8^+^ T cells again from the cocultures using the Miltenyi CD8^+^ T cell magnetic isolation kit. The CD8^+^ T cells were then stained with CFSE (Invitrogen, C34570). 140,000 stained CD8^+^ T cells were then added to the tumor cells in the 8 well plate. Using a high-content dual spinning disk confocal microscope, Yokogawa CellVoyager CQ3000, and time-series was performed over 13 h using a 60X/1.2 NA W objective lens, acquiring both brightfield and fluorescence images (488 nm excitation, BP 525/50 emission). Tumor cell death was identified in transmission images by rounded morphology (dead cells), whereas live tumor cells remained elongated. The percentage of tumor cell killing was calculated as %killing = (dead cell count/(total tumor cell count*CD8count))*100. A negative control of tumor cells alone was included to assess the spontaneous cell death in the absence of CD8^+^ T cells. The area under the curve (AUC) of tumor cell killing over time was then calculated to quantify the overall cytotoxic activity of CD8^+^ T cell for each condition. Each data point represents a tile in the well, with each tile composed of 10*10 fields.

#### Incucyte CD8^+^ T cell tumor cell killing assay

B cells were isolated from TDLNs of mammary tumor bearing mice using Miltenyi B Cell magnetic isolation kit (Miltenyi, #130–104-443). CD8^+^ T cells were isolated from spleens of FVB mice using a Miltenyi CD8^+^ T cell magnetic isolation kit (Miltenyi, #130–104-075) and stimulated using an anti-CD3 antibody (BD, # 553057). B cells and CD8 T cells were cocultured at a 1:2 ratio for 48 h. Tumor cells were labeled with Incucyte Cytolight Rapid Red dye (Sartorius, #4706) at a final concentration of 0.5 μM and seeded at 4,000 cells per well in a 96-well plate overnight. The next day (corresponding to 48 h of B/T coculture), the medium from the tumor cell wells was removed, and Annexin V dye (Sartorius, #4642) was added at a 1:200 dilution. Immune cells were counted and added to the wells at a 1:10 ratio (40,000 immune cells per well). Tumor cells positive for Annexin V (green) were quantified using Incucyte software. Results are presented as the area under the curve (AUC) representing the cumulative annexin V staining on tumor cells overtime. Each point an individual mouse and the graph combines data from two independent experiments.

#### Differentiation of bone marrow derived macrophages (BMDM)

Femur bones were acquired from female FVB mice of 10–12 weeks. Epiphyses of the femur were removed, and bone marrow was isolated by flushing the femurs with PBS. Erythrocytes were removed using ACK lysing buffer for 3 min. Bone marrow cells were then placed in macrophage differentiation media consisting of RPBMI-1640 (Gibco, 11875–119), 10% FBS (Cytiva, #SH30070.03), 100 U/mL of Penicillin 100 μg/mL of Streptomycin (HyClone,# SV30010), 1X NeAA (Gibco, #11140050), 25 mM HEPES (Gibco, #15630080), 1 mM NaPyruvate (Gibco, #11360070), and supplemented with 30% L-cell media which was isolated from L-cell culture supernatant which contains M-CSF. Bone Marrow cells were then placed onto 10 cm low attachment tissue culture plates (Corning, #4615) at 4e6 cells per plate. On day 4 of BMDM culture, differentiation media was added to the plates. Day 7 of culture BMDMs were isolated from the plate and utilized for downstream experiments.

#### Co-culture of B cells and macrophages

B cells were isolated by magnetic sorting (Miltenyi, Pan B Cell Isolation Kit II mouse #130–104-443) from TDLNs of CD40 treated or isotype treated mice. BMDM were differentiated according to BMDM differentiation protocol. B Cells and BMDM were co-cultured in a 2:1 ratio for 24–48 h. Cells were co-cultured in RPMI-1640 (Gibco, #11875–119) supplemented with 10% FBS (Cytiva, #SH30070.03), 100 U/mL of Penicillin 100 μg/mL of Streptomycin (HyClone,# SV30010), 1X NeAA (Gibco, #11140050), 25 mM HEPES (Gibco, #15630080), 1 mM NaPyruvate (Gibco, #11360070), 100 ng/mL IL4 (Biolegend,#574306), 2 μg/mL BAFF (Acro Biosytems,#BAF-M521Y), 0.05 mM 2-Mercaptoethanol (Gibco, #21985023) freshly added to the medium. After co-culture cells were taken for flow cytometry analysis and the supernatant was taken for downstream assays.

#### ELISA

Cells from co-culture were centrifuged at 400×*g* for 5 min, and the supernatant was collected and stored at −80C. Samples were tested for CXCL9 levels using a Mouse CXCL9/MIG DuoSet ELISA (R&D Systems, #DY492–05). Uncoated nunc maxisorb ELISA plates (Biolegend, #423501) were coated with CXCL9 capture antibody and incubated overnight in 4C. The plate was washed with wash buffer and then blocked using blocking buffer for 1 h. Plates were washed 2x in washing buffer. Both standards as well as supernatant from co-culture were added to the plate and incubated for 2 h. Plates were washed 2x with washing buffer. Detection antibodies were added, and plates were incubated for 2 h. Plates were washed 2x with washing buffer. Streptavidin-HRP were added to the plate and incubated for 20 min in the dark. Plates were washed 2x with washing buffer. Substrate solutions were added to the plate and incubated for 20 min in the dark. Stop solution was then added and the plate was read at 450 nm on a microplate reader.

#### CD8 T cell migration assay

CD8^+^ T cells were isolated from FVB mouse spleen by magnetic separation (Miltenyi CD8^+^ T cell isolation kit, 130–104-075). Isolated CD8^+^ T cells were resuspended in RPMI +2% FBS. 200.000 cells were seeded on top of a transwell of 3 μm pore size (Sigma-Aldrich, PTSP24H48). 500 μL of migration media, consisting of RPMI, 2% FBS, and 25% of culture supernatant isolated from B cell-macrophage cocultures, were placed on the bottom of the transwell in a 24 well plate. 500 μL of RPMI with 10% FBS were used as positive control of maximum migration and 500 μL of RPMI with 2% FBS were used as basal mgration. Migration was allowed for 24 h in the incubator at 37°C, 5% CO2. After 24 h the cells on the bottom of the plate were collected, stained with LIVE/DEAD Fixable Aqua (ThermoFisher, L34957) and acquired with the high-throughput sampler of a BD FACS Symphony A3, with defined acquisition volumes. Live cells were counted using the CountBright counting beads (Invitrogen C36950) following manufacturer’s instruction. The number of migrating cells were normalized to the basal migration.

#### RNA seq

RNA-seq analysis mRNA was isolated using the Qiagen RNeasy kit according to manufacturer protocol. mRNA quality was assessed using the Agilent TapeStation 2200 and libraries for mRNA-seq made using total RNA in conjunction with TruSeq Stranded mRNA Library Prep kit (Illumina, #20020595). Libraries were sequenced by the Salk Institute Next Generation Sequencing facility and UNC High Throughput Sequencing Facility. Samples sequenced by UNC High Throughput Sequencing Facility were performed on the Illumina HiSeq 2000/2500 sequencer. Samples sequenced by the Salk Institute Next Generation Sequencing facility were performed on the Illumina NextSeq2000 sequencer.

Fastq files obtained from the sequencing runs were aligned using the STAR aligner algorithm using the mus musculus mm10 genome as a reference.^141^ Samtools were then utilized on the resulting BAM files to organize and index them. Picard was used to access quality control on the BAM files. Read counts from transcripts were assessed using the Salmon pipeline.^152^ Genes that displayed low read on the scale of less than 10 and those with no read counts across samples were removed from the dataset. Gene counts from the Salmon pipeline were then submitted to upper quartile normalization,^153^ followed by log2 transformation through Cluster 3.0. A matrix was then established for statistical analyses by median centering the log2 transformed values. To perform hierarchical clustering on both human and mouse datasets cluster 3.0 was used,^143^ visualization of hierarchical clustering was performed through Java Tree View. All analysis that required mouse genes to be converted to human genes for analyses in human datasets utilized Biomart for the gene conversion.^154^ For analyses that involved the use of gene signatures, the median expression of all the genes within the signature were utilized to measure expression of the gene signature.^155^ Published mouse mammary tumor RNA-seq data is available at GSE124821.^13^ New samples in this report are deposited at GSE276004.

#### Single cell RNA-Seq

Single cell suspensions from tumor tissues for scRNA seq were generated by using Miltenyi’s mouse dissociation kit (Miltenyi, 130–096-730) and a gentleMACS octo tissue dissociator(Miltenyi, 130–096-427). KPB25Luv CD40ag scRNA seq was prepared using 10x Genomics single cell 3′ reagent kit v2 following 10x Genomics single cell 3′ reagent kit v2 user manual (Manual Part # CG00052 Rev A). KPB25Luv aCD19/CD40ag scRNA seq was prepared using 10x Genomics singe cell 5′ reagent kit v2 following 10x Genomics singe cell 5′ reagent kit v2 user manual (Manual Part# CG000331 Rev F). Single cell GEMs were created using a 10x Genomics Chromium controller. KPB25Luv CD40ag single cell library was created using Chromium Single Cell 3′ Library & Gel Bead Kit v2 (10x Genomics, PN-120237) and i7 Multiplex Kit (10x Genomics, PN-120262). KPB25Luv single cell library was created using a Library Construction Kit (10x Genomics, PN-1000190) and a Dual Index Kit TT Set A (10x Genomics, PN-1000215). KPB25Luv CD40ag scRNA seq libraries were sequenced using an Illumina HiSeq 4000 as 2 × 150 paired end reads. KPB25Luv aCD19/CD40ag scRNA seq libraries were sequenced using an Illumina Next seq2000 as paired end reads. The resulting Fastq files from sequencing KPB25Luv CD40ag single cell were then processed through 10x Genomics Cell Ranger version 3.0.2. KPB25Luv aCD19/CD40ag Fastq files were processed through Cell Ranger version 6.1.1. Cell Ranger was used for demultiplexing the single cell dataset along with barcode and UMI counting. Sequence alignment was performed using mus musculus mm10 dataset. Further Details on how Cell Ranger is used can be found https://www.10xgenomics.com/support/software/cell-ranger/latest/getting-started/cr-what-is-cell-ranger. Published mouse mammary tumor single cell RNA-seq data can be found on GEO datasets GSE136206.^13^ New single cell sequencing data from this study is deposited at GSE276006.

After data processing through Cell Ranger, the outputs were analyzed utilizing the R-package Seurat Version 5.0.0. Preprocessing of Cell Ranger outputs followed the Seurat pipeline. First cells were filtered out of the dataset based on their doublet quality, high mitochondrial gene count, and low number of expressed genes. The dataset was then submitted to log-normalization and variable genes among cells were elucidated through their standard deviation from the average expression of those genes. After variable gene identification the data was scaled and PCA analysis was performed. Clusters were created using shared nearest neighbors and reduction was performed based on PCA, which were then both utilized for UMAP analysis. To perform analyses between different processed single cell datasets Harmony Integration was employed and subsequent processing of the integrated dataset followed Seurat’s pipeline.^140^ Single cell mRNA signatures for cell types were derived from comparing clusters using the Seurat FindAllMarkers function.^140^ Immune cell phenotypes were identified as in prior work,^13^ memory-like B cells were identified using markers that are conserved in human memory B cells.^84,121,156^ Single cell RNA seq cell to cell communication predication analysis was performed using the R-package CellChat Version 2.1.0. Preprocessing, dataset creation, and analysis protocol were conducted following the CellChat protocol.^103^

#### Human dataset analysis

For analysis in human patients with breast cancer, we used the following published datasets: Calg40603/DbGAP phs001863.v1.p1,^67,70^ Ncbi Geodatasets study GSE32646 on ER-negative breast tumors,^68^ and NCT01560663/Madrid.^69^ For signature analysis, mouse scRNA-seq results from Seurat’s FindAllMarkers function was used to identify the top 100 markers for each immune cell cluster/subset. Mouse gene nomenclature was converted to human gene symbols and then each human tumor was scored for the median expression of the signature. For the proliferative CD8^+^ T cells, all analysis were done with proliferation genes removed, noting that proliferation-linked genes were identified by presence in annotated proliferation gene signatures in mSigDB.^83,157^ For supervised analysis, we separated patient tumors into PCR or no PCR and then performed a two-tailed unpaired student *t* test to determine the extent of association of a signature with clinical outcome. Likewise, we performed linear regression analysis in graphpad prism to identify the degree of correlation between two gene signatures.

#### Signature scoring and differential expression

Significant changes in expression of gene expression in bulk RNA-seq datasets was detected by DESEQ2 and Benjamini Hochburg testing.^158,159^ In single cell data, the Seurat FindAllMarkers was used to identify differentially expressed genes based on Wilcoxon Rank Sum statistics.^140,160^ Conventional gene set enrichment analysis was used to detect enrichment of memory B cell signatures.^83^ For scoring gene signature expression levels in the main figures, signatures levels were scored using single sample GSEA and GenePattern.^83,97^ In supplemental statistics, we used median expression of signature genes as a second signature expression level scoring metric.^155^

### QUANTIFICATION AND STATISTICAL ANALYSIS

All modes of quantification and statistical analysis can be found in the supplemental statistics file (Data S1) along with the corresponding method detail and figure legends.

## Supplementary Material

Video S1

Video S2

1

SUPPLEMENTAL INFORMATION

Supplemental information can be found online at https://doi.org/10.1016/j.celrep.2026.117957.

## Figures and Tables

**Figure 1.**
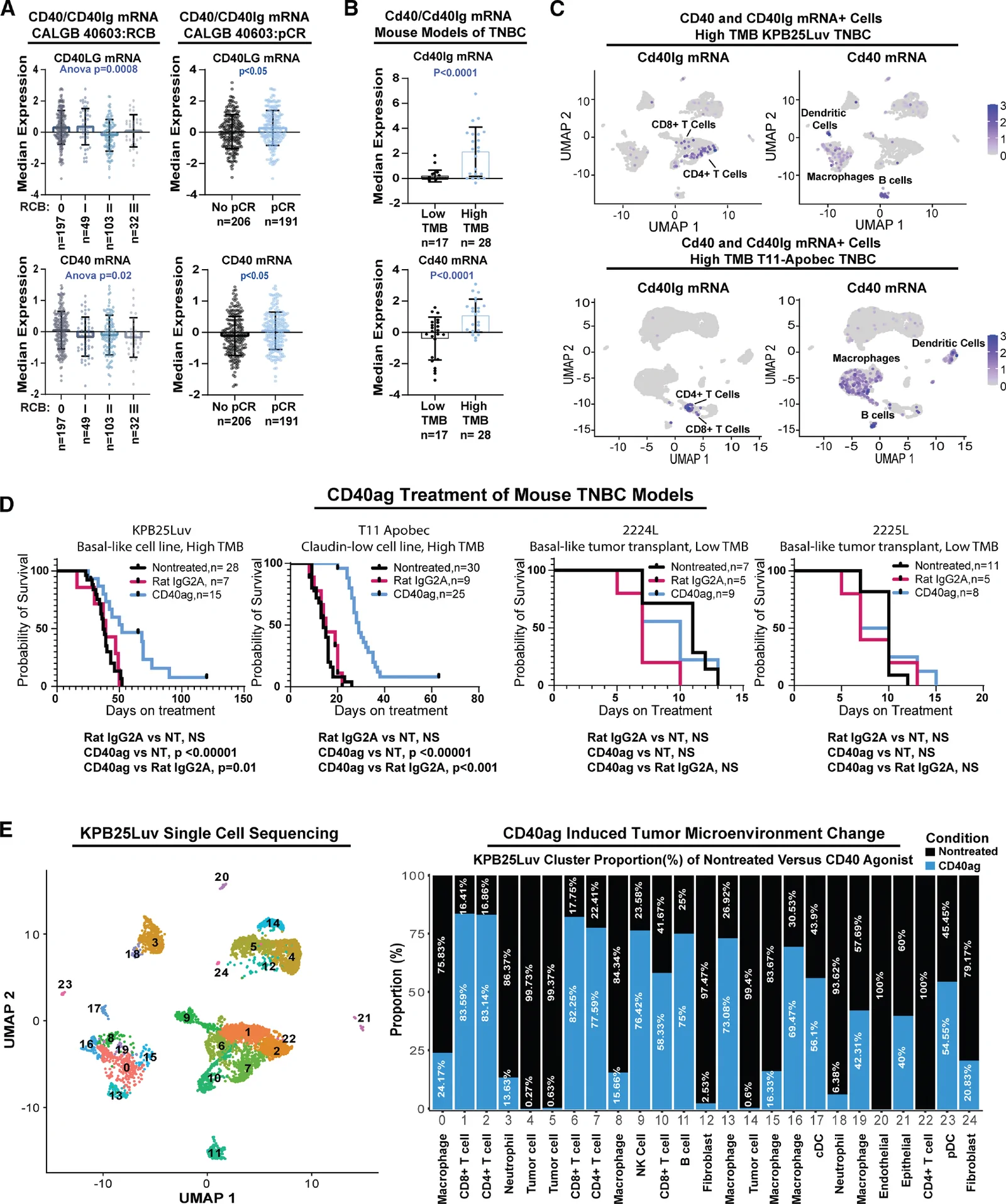
The CD40 pathway is active in mouse and human triple-negative breast cancers (A) CD40 and CD40lg mRNA expression levels in a published bulk RNA-sequencing dataset of human TNBCs^67^; Both residual cancer burden (RCB) and pathological complete response (PCR) status is based on outcomes in a neoadjuvant chemotherapy dataset from the CALG 40603 clinical trial.^67,70^ For PCR, *p* value is calculated by DESEQ2 analysis. For RCB, *p* value is calculated by a one-way ANOVA. (B) CD40 and CD40lg mRNA expression levels in a published bulk RNA-sequencing dataset (GSE124821) of primary tumors from high- or low-TMB TNBC mouse models.^13^ *p* value is calculated by DESEQ2 analysis. (C) Feature plot of KPB25Luv and T11-Apobec nontreated tumors showing cells expressing CD40 or CD40lg at 5 mm size. Data are published and available at GSE136206.^13^ (D) Survival of KPB25Luv, T11-Apobec, 2224L, and 2225L TNBC mouse models treated with Rat IgG2A isotype control or CD40ag. Respective comparisons and *p* value were calculated by Kaplan-Meier analysis. NS = not significant. Black blocks on the plot are censored events. (E) Uniform Manifold Approximation and Projection (UMAP) projection of single-cell sequencing of KPB25Luv tumors collected at 9 days after reaching a diameter of 5 mm with or without 3 doses of CD40ag. UMAP cluster compares the proportion of cells from KPB25Luv CD40ag-treated tumors and nontreated tumors.

**Figure 2.**
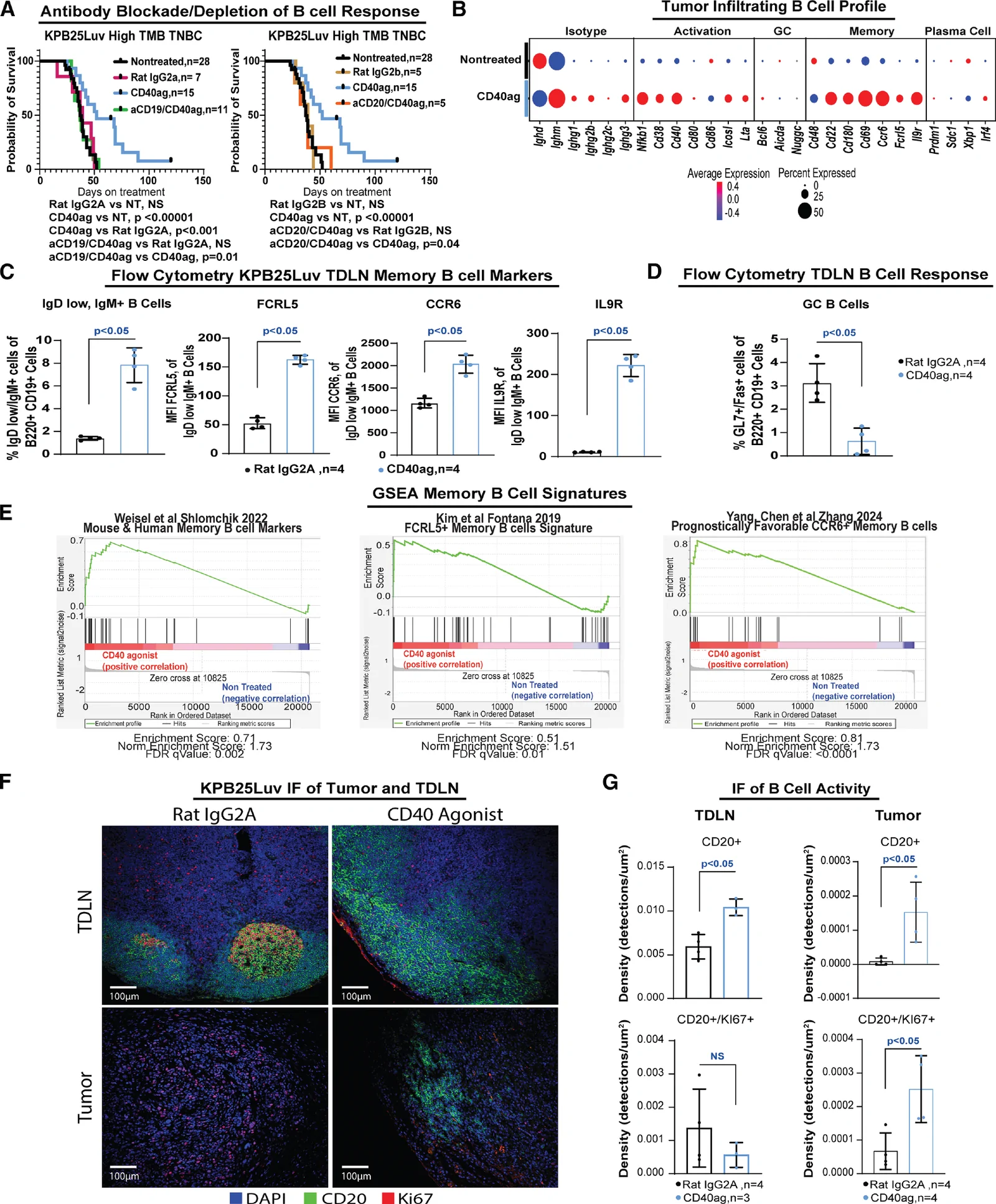
CD40 agonist treatment induces an anti-tumor B Cell response in two high TMB TNBC mouse models (A) Survival of mice implanted with KPB25Luv treated with either Rat IgG2A isotype control antibody, Rat IgG2B isotype control antibody, CD40ag antibody, CD40ag delivered concurrently with anti-CD19 inhibitory antibody, or CD40ag delivered concurrently with anti-CD20 depleting antibody. Kaplan-Meier analysis used comparisons to the CD40 agonist treatment group to statistically test survival differences. NS = not significant. Black blocks on plot are censored events. (B) Dot plot showing the expression level along with proportion of cells differentially expressing key markers of B cell phenotype and potential function. Comparisons are between CD40ag and nontreated KPB25Luv tumor-infiltrating B cells. Dot size relates to the proportion of cells with gene expression as indicated in the key; dot color relates to expression level as indicated by the color bar. Wilcoxon rank statistics are provided in the supplemental statistics file. (C) Flow cytometry cellular quantification of IgD (low) IgM+ B cells gated on B220^+^ CD19^+^ cells in addition to FCRL5, CCR6, and IL9R mean fluorescence intensity (MFI) in live B220^+^ CD19^+^ IgM + IgD low B cells from the TDLNs of KPB25Luv-bearing mice treated with CD40ag antibody or rat IgG2A isotype control antibody. Statistics and *p* value are calculated by a two-tailed unpaired Mann-Whitney test. (D) Flow cytometry shows the cellular proportion quantification of FAS^+^ GL7^+^ B cells gated on B220^+^ CD19^+^ B cells from TDLNs of KPB25Luv-bearing mice treated with CD40ag antibody or rat IgG2A isotype control antibody. Statistics and *p* value are calculated by a two-tailed unpaired Mann-Whitney test. (E) Statistical Gene Set Enrichment Analysis^83^ of both human and mouse memory B cell gene signatures^46,84,85^ applied to KPB25Luv scRNA-seq B cell cluster. Comparisons are made between CD40ag-treated and non-treated tumors. (F) Immunofluorescence staining for DAPI (blue), CD20 (green), and Ki67 (red) in Rat IgG2A isotype control or CD40Ag-treated KPB25Luv tumors along with TDLNs. Scale bars represent 100 μm. (G) QuPath quantification of tumor and TDLN images for CD20^+^ positive cells along with CD20^+^ Ki67^+^ cells in Rat IgG2A isotype control and CD40ag-treated groups. Whole tumor tissue area was used for quantification, along with only B cell follicles within TDLNs. Statistics and *p* value are calculated by a two-tailed unpaired Mann-Whitney test.

**Figure 3.**
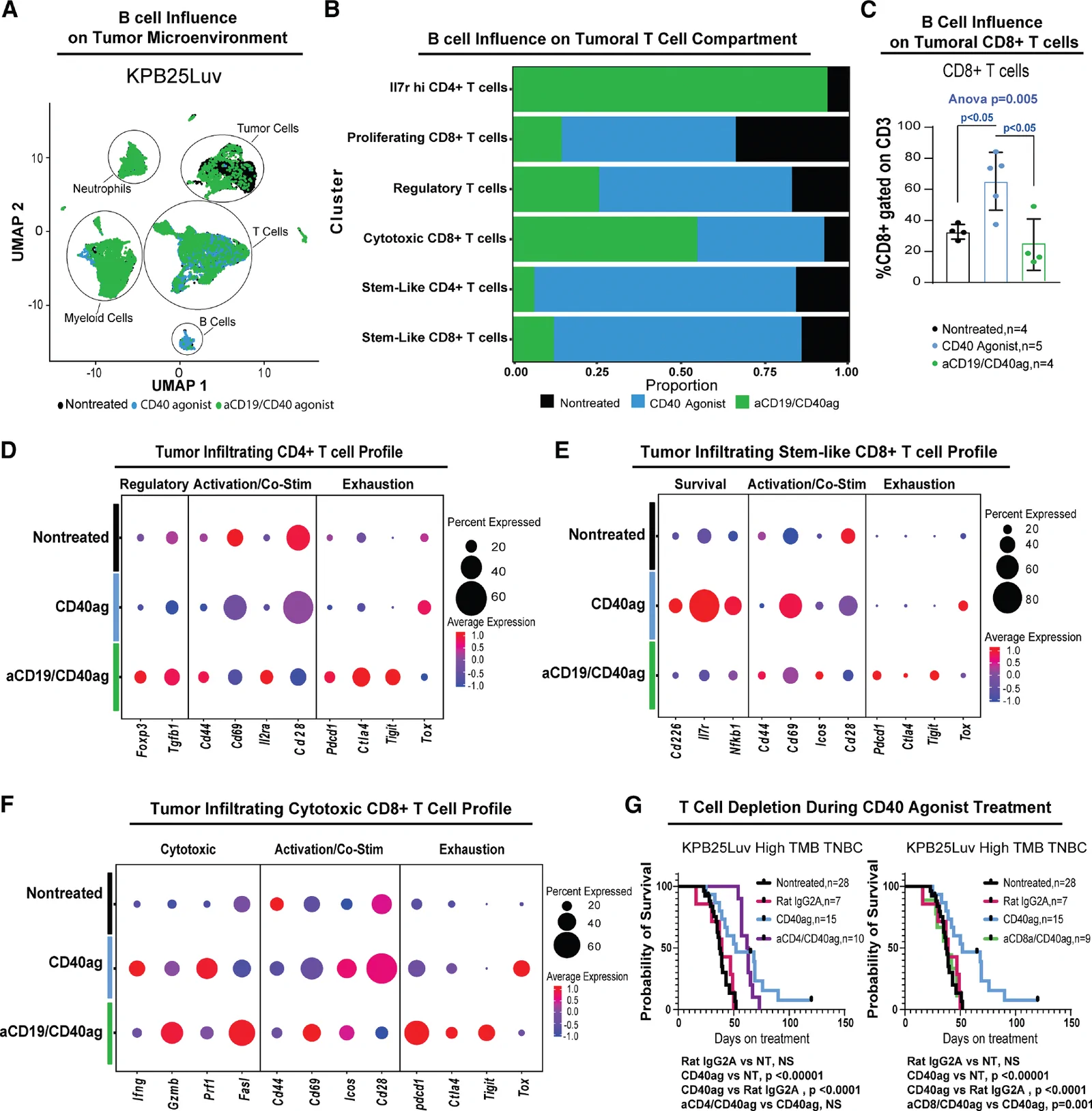
CD40 agonist-driven B cell responses promote anti-tumor CD8^+^ T cell activity (A) UMAP of nontreated (black), CD40 agonist (blue), and CD40 agonist delivered concurrently with anti-CD19 antibodies (green) of KPB25luv tumors. Treated tumors were collected and sequenced on day 9 following three doses of CD40ag or CD40ag delivered with anti-CD19 antibody treatments. Non-treated tumors are time-matched for 9 days after tumor diameter reaches 5 mm. (B) scRNA-seq-derived T cell cluster proportions in nontreated, CD40ag treated, or CD40ag delivered with anti-CD19 treated KPB25Luv tumors. (C) Flow cytometry quantification of CD8^+^ T cell proportion of KPB25Luv tumors of either nontreated, CD40 agonist treated, or CD40 agonist delivered with anti-CD19 treated KPB25Luv tumors. Specimens were taken after 9 days and 3 antibody doses. Non-treated are time-matched to days after tumor diameter reaches 5 mm. Statistics and *p* value are the result of a one-way ANOVA, followed by Tukey’s multiple comparisons test. (D) scRNA-seq dot plot of CD4^+^ T cell clusters depicting key CD4^+^ T cell markers and differentially expressed genes based on treatment status of KPB25Luv tumors. Differential expression calculated by Seurat FindAllMarkers. Dot size relates to the proportion of cells with gene expression as indicated in the key; dot color relates to expression level as indicated by the color bar. See supplemental statistics for individual genes. Wilcoxon rank statistics are provided in the supplemental statistics file. (E) scRNA-seq dot plot of stem-like CD8^+^ T cell cluster cells depicting key T cell markers and differentially expressed genes based on treatment status of KPB25Luv tumors. Differential expression was calculated using Seurat’s FindAllMarkers. Dot size relates to the proportion of cells with gene expression as indicated in the key, dot color relates to expression level as indicated by the color bar. See supplemental statistics for individual genes. Wilcoxon rank statistics are provided in the supplemental statistics file. (F) scRNA-seq dot plot of cytotoxic CD8^+^ T cell cluster cells depicting key T cell markers and differentially expressed genes based on treatment status of KPB25Luv tumors. Differential expression was calculated using Seurat FindAllMarkers. Dot size relates to the proportion of cells with expression as indicated in the key; dot color relates to expression level as indicated by the color bar. See supplemental statistics for individual genes. Wilcoxon rank statistics are provided in the supplemental statistics file. (G) Survival of mice implanted with KPB25Luv tumors treated with either Rat IgG2A isotype control, CD40 agonist, CD40 agonist concurrently with anti-CD4 antibodies (anti-CD4 is depleting^13^) or with anti-CD8 antibodies (anti-CD8 is depleting^13^). Kaplan-Meier analysis used comparisons to the CD40 agonist treatment group to statistically test survival differences. See prior publication for verification of anti-CD4 and anti-CD8 depleting function.^13^ Black blocks on plot are censored events.

**Figure 4.**
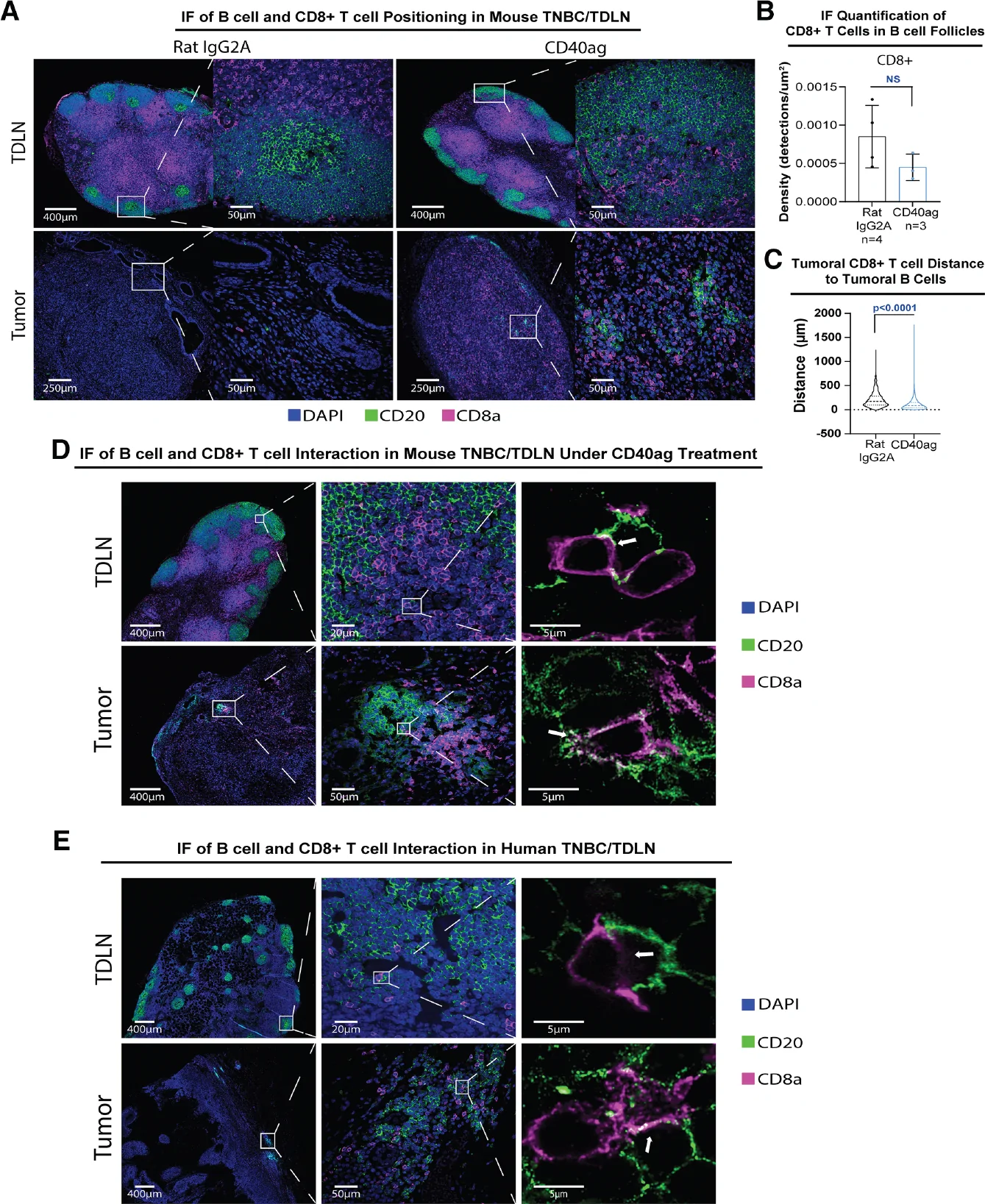
B cells and CD8^+^ T cells are colocalized and exhibit physical interaction in CD40 agonist-treated mouse TNBCs and non-treated human TNBCs (A) Immunofluorescence staining for DAPI (blue), CD20 (green), and CD8a (magenta) in Rat IgG2A isotype control or CD40ag-treated KPB25Luv tumors along with TDLNs. Scale bars for TDLN from left to right represents 400 μm (zoomed out), and 50 μm (zoomed in). Scale bars for tumors from left to right represent 250 μm (zoomed out), and 50 μm (zoomed in). (B) Qupath quantification of CD8^+^ cell density within B cell follicles of both Rat IgG2A isotype control and CD40 agonist-treated KPB25Luv TDLNs. Quantification was only performed on B cell follicles. Statistics and *p* value are calculated by a two-tailed unpaired Mann-Whitney test. (C) Qupath analysis of distance between CD8^+^ cells and CD20^+^ cells within Rat IgG2A isotype control and CD40 agonist-treated KPB25Luv tumors, using cells as independent measures. Whole-tumor tissue area was used for quantification. Statistics and *p* value are calculated by a two-tailed unpaired Mann-Whitney test; *n* = 3 tumors were used for Rat IgG2A isotype controls, and *n* = 5 tumor were used for CD40ag-treated groups. (D) Immunofluorescence of colocalized signal between a CD20^+^ cell and a CD8^+^ cell in a CD40Ag-treated KPB25Luv TDLN along with a CD40Ag-treated KPB25Luv tumor. The white arrow indicates the interface of the cell interaction. Scale bars for TDLNS from left to right represent 400 μm, 20 μm, and 5 μm. Scale bars for tumors from left to right represent 400 μm, 50 μm, and 5 μm. (E) Immunofluorescence of colocalized signal between a CD20^+^ cell and a CD8^+^ cell within the B cell follicle of a human TNBC patient TDLN along with the primary tumor. The white arrow indicates the interface of the cell interaction. Scale bars for TDLNS from left to right represent 400 μm, 20 μm, and 5 μm. Scale bars for tumors from left to right represent 400 μm, 50 μm, and 5 μm.

**Figure 5.**
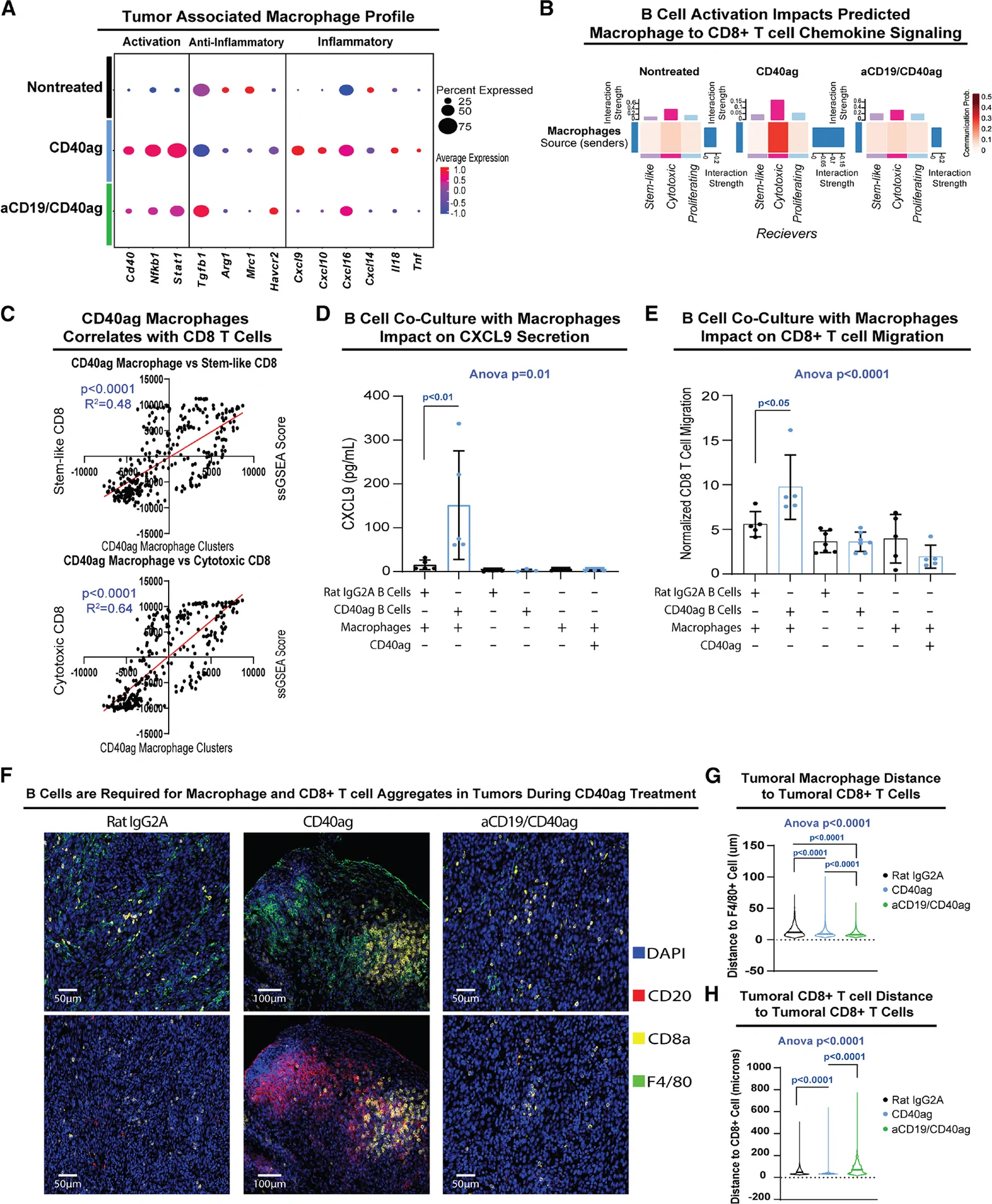
CD40 agonist-driven B cell responses elicit macrophage-mediated CD8^+^ T cell chemotaxis and organize CD8^+^ T cell aggregates in the tumor (A) Dot plot of key macrophage markers and differentially expressed genes based on treatment status of macrophage scRNA-seq clusters of KPB25Luv tumors. Differential expression calculated by Seurat FindAllMarkers. Dot size relates to the proportion of cells with gene expression as indicated in the key; dot color relates to expression level as indicated by the color bar. See supplemental statistics for individual genes. Wilcoxon rank statistics are provided in the supplemental statistics file. (B) scRNA-seq CellChat Heatmap of CXCL pathway predicted interaction strength between Macrophage and CD8^+^ T cell clusters of KPB25Luv nontreated, CD40 agonist, and CD40 agonist delivered with anti-CD19-treated tumors. (C) Correlation Plot of stem-like CD8 T cell and cytotoxic CD8^+^ T cell gene signatures with macrophage gene signature, derived from scRNA-seq clusters of KPB25Luv tumors, in the mouse mammary tumor dataset,^13^ GSE124821. R-squared and *p* value calculated by linear regression analysis. (D) CXCL9 ELISA of supernatant from 48-h coculture of BMDMs and B cells isolated from TDLNs of Rat IgG2A isotype control-treated or CD40 agonist-treated KPB25Luv tumor-bearing mice. Statistics and *p* value are calculated by one-way ANOVA, followed by Tukey’s multiple comparisons test. (E) Quantification of CD8^+^ T cell migration through a Boyden chamber using supernatant from 48-h coculture of BMDMs and B cells isolated from TDLNS of Rat IgG2A isotype control-treated or CD40 agonist-treated KPB25Luv tumor-bearing mice. Migration is normalized to CD8^+^ T cells alone with unconditioned media. Statistics and *p* value are calculated by a one-way ANOVA test, followed by Tukey’s multiple comparisons test. (F) Immunofluorescence for DAPI (blue), F4/80 (green), CD20 (red), and CD8a (yellow) of Rat IgG2A isotype control, CD40 agonist, or CD40 agonist/anti-CD19 treated KPB25Luv tumors. Top and bottom images are from parallel sections of the same tumor area. Scale bars from left to right represent 50 μm, 100 μm, and 50 μm. (G) QuPath distance quantification of F4/80^+^ cells and CD8^+^ cells in Rat IgG2A isotype control, CD40 agonist, or CD40 agonist/anti-CD19 treated KPB25Luv tumors, using cells as independent measures. Whole tumor tissue area was used for quantification. Statistics and *p* value are calculated by a one-way ANOVA test, followed by Tukey’s multiple comparisons test; *n* = 3 tumors were used for Rat IgG2A isotype controls, *n* = 3 tumor were used for CD40ag-treated groups, and *n* = 3 tumors were used in the CD40ag delivered with anti-CD19 groups. (H) QuPath quantification of CD8^+^ cell distances to one another in Rat IgG2A isotype control, CD40 agonist, or CD40 agonist/anti-CD19 treated KPB25Luv tumors, using cells as independent measures. Whole tumor tissue area was used for quantification. CD8^+^ cell distance from each CD8^+^ cell is calculated using *n* = 4 tumors for Rat IgG2A isotype controls, *n* = 7 tumor were used for CD40ag-treated groups, and *n* = 4 tumors for CD40ag delivered with anti-CD19 groups. Statistics and *p* value are calculated by a one-way ANOVA test, followed by Tukey’s multiple comparisons test.

**Figure 6.**
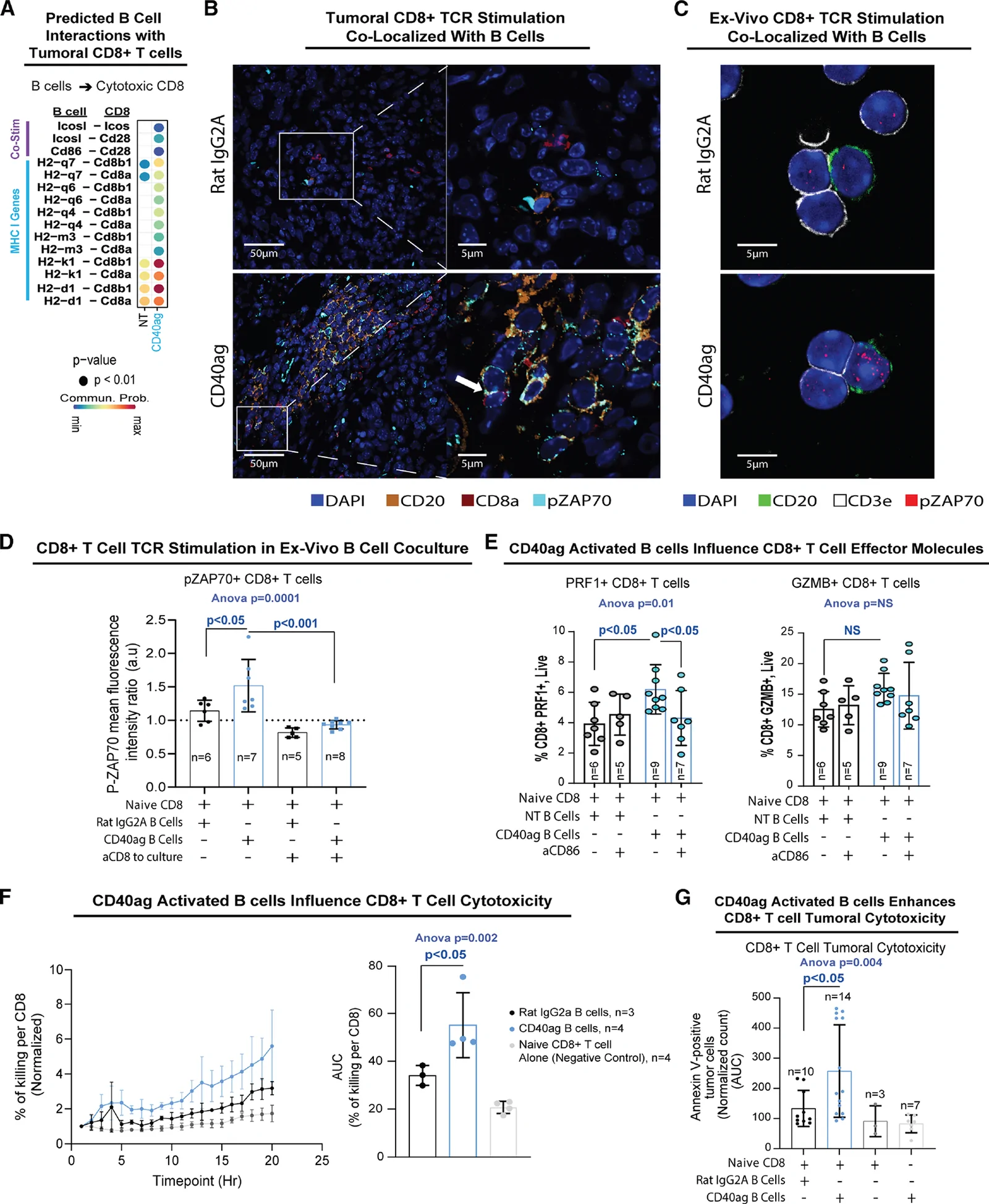
CD40 agonist-driven B cell responses activate CD8^+^ T cells to induce tumor cell killing (A) scRNA-seq CellChat dot plot of key B cell interactions with cytotoxic CD8^+^ T cells within CD40 agonist-treated and nontreated KPB25Luv tumors. The dot color and size are indicative of the calculated communication probability. The respective *p* values are computed from a one-sided permutation test. (B) Immunofluorescence of DAPI (blue), CD8a (red), phosphorylated Zap70 (cyan, pZap70), and CD20 (orange) from Rat IgG2A isotype control and CD40 agonist-treated KPB25Luv tumors. Scale bars from left to right represent 50 μm (zoomed out) and 5 μm (zoomed in). (C) Immunofluorescence of DAPI (blue), CD3E (white), phosphorylated Zap70 (red, pZap70), and CD20 (green) in a B cell-CD8^+^ T cell co-culture. B cells were isolated from Rat IgG2A isotype control and CD40 agonist-treated KPB25Luv TDLNs. CD8^+^ T cells were isolated from spleens of tumor-naïve mice. Scale bars represent 5 μm. (D) Microscopy quantification of the pZAP70 mean fluorescence intensity (MFI) in T cells across the different coculture conditions of CD8^+^ T cells with B cells from tumor-bearing mice treated with either isotype control or CD40 agonist, with or without *in vitro* aCD8 treatment. PZAP70 fluorescence intensity was analyzed using Fiji/ImageJ.^111^ At least 50 nuclei were analyzed per image, and the MFI of pZAP70 was quantified on the original images after ROI definition based on DAPI staining. The average fluorescence intensity of pZAP70 was then calculated for each image. At least 100 CD8^+^ T cells were analyzed per biological replicate. Values were normalized to the background fluorescence obtained from samples processed without primary anti-pZAP70 antibody staining. The line at 1 indicates the normalized background baseline. Each point on the graph represents the average value from a single image. Images were acquired from three independent biological replicates (*n* = 3). Statistics and *p* values were calculated by one-way ANOVA test, followed by Tukey’s multiple comparisons test. (E) Flow cytometric evaluation of Perforin 1 or Granzyme B in CD8^+^ T cells following a B cell *ex vivo* co-culture using B cells isolated from either nontreated mice or CD40 agonist-treated KPB25Luv TDLNs. Anti-CD3 antibody was used to stimulate naive CD8 T cells. Anti-CD86 antibodies were added to *in vitro* to block CD86 activation of the CD28 pathway in CD8^+^ T cells. Statistics and *p* value are calculated by a one-way ANOVA test, followed by Tukey’s multiple comparisons test. (F) Live imaging quantification of KPB25Luv tumor cell death over time. CD8^+^ T cells were previously cultured with B cells isolated from the TDLNs of tumor-bearing mice injected with either isotype control or CD40 agonist. The CD8^+^ T cells were then isolated from the co-culture and stained with CFSE (green dye). Anti-CD3 antibody was used to stimulate naive CD8^+^ T cells. These cells were co-cultured with the adherent KPB25Luv tumor cells to assess the cytotoxic potential of CD8^+^ T cells. Tumor cell death was identified in transmission images by rounded morphology (dead cells), whereas live tumor cells remained elongated. The percentage of tumor cell killing was calculated as %killing = (dead cell count/(total tumor cell count*CD8count))*100. A negative control of tumor cells alone was included to assess the spontaneous cell death in the absence of CD8(+) T cells. The area under the curve (AUC) of tumor cell killing over time was then calculated to quantify the overall cytotoxic activity of CD8^+^ T cells for each condition. Each data point represents a tile in the well, with each tile composed of 10*10 fields. Statistics and *p* value are calculated by a one-way ANOVA test, followed by Tukey’s multiple comparisons test. (G) Incucyte quantification of tumor cells positive for annexin V staining from an *ex vivo* coculture of KPB25Luv tumor cells with CD8^+^ T cells activated by anti-CD3 antibodies that were previously cultured with B cells isolated from KPB25Luv tumor-bearing mice treated with either Rat IgG2A isotype control or CD40 agonist. Data are expressed as AUC, representing the cumulative annexin V staining on tumor cells overtime. Each point is an individual mouse, and the graph combines data from two independent experiments. Statistics and *p* value are calculated by a one-way ANOVA test, followed by Tukey’s multiple comparisons test.

**KEY RESOURCES TABLE:** 

REAGENT or RESOURCE	SOURCE	IDENTIFIER

Antibodies		

Anti-mouse PD1 (clone RMP1-14)	Bioxcel	Cat: BE0146; RRID: AB_10949053
Anti-mouse CD40 (clone FGK45)	Leinco	Cat: F1195; RRID: AB_2830170
Anti-mouse CD4 (clone GK1.5)	Leinco	Cat: C2838; RRID: AB_2829596
Anti-mouse CD8a (clone Ly 2)	Leinco	Cat: C2848; RRID: AB_2829603
Anti-mouse CD19 (clone 1D3)	Leinco	Cat: C2849; RRID: AB_2829604
Anti-mouse CD20 (clone SA271G2)	Biolegend	Cat: 152104; RRID: AB_2629619
Anti-mouse CD86 (clone GL-1)	Bioxcel	Cat: BE0025; RRID: AB_1107678
Anti-mouse CD8b (clone 53-5.8)	Bioxcel	Cat: BE0223; RRID: AB_2687706
Rat IgG2b Isotype Control (clone 1-2)	Leinco	Cat: I-1034; RRID: AB_2737522
Rat IgG2a Isotype Control (clone 1-1)	Leinco	Cat: I-1177; RRID: AB_2737530
Anti-mouse CD16/CD32 (clone 2.4G2)	BD Biosciences	Cat: 553141; RRID: AB_394656
Anti-mouse CD3e (clone 145-2C11)	BD Biosciences	Cat: 553057; RRID: AB_394590
Anti-mouse CD45, BV605 (clone 30-F11)	Biolegend	Cat: 103139; RRID: AB_2562341
Anti-mouse B220, BV785 (clone RA3-6B2)	Biolegend	Cat: 103246; RRID: AB_2563256
Anti-mouse CD19, BV421 (clone 1D3)	Biolegend	Cat: 152421; RRID: AB_3106188
Anti-mouse CD19, PE-CF594 (clone 1D3)	BD Biosciences	Cat: 562291; RRID:AB_11154580
Anti-mouse CD20, AF647 (clone GOT214A)	BD Biosciences	Cat: 568251; RRID: AB_3684140
Anti-mouse IgD, APC-H7 (clone 11-26c.2a)	BD Biosciences	Cat: 565348; RRID:AB_2739201
Anti-mouse IgM, BV605 (clone RMM-1)	Biolegend	Cat: 406523; RRID:AB_2563358
Anti-mouse Il9r, PE (clone S18011E)	Biolegend	Cat: 158704; RRID:AB_2860743
Anti-mouse/human CD11b, Spark UV 387 (clone M1/70)	Biolegend	Cat: 101292; RRID:AB_3716883
Anti-mouse F4/80, PE (clone BM8)	Biolegend	Cat: 123109; RRID:AB_893498
Anti-mouse Ly-6G, PE/Cy5 (clone 1A8)	Biolegend	Cat: 127671; RRID:AB_2904289
Anti-mouse CD206, FITC (clone C068C2)	Biolegend	Cat: 141703; RRID:AB_10900988
Anti-mouse/human CXCL9 (Polyclonal)	Abcam	Cat: ab202961, RRID: AB_3105888
Anti-mouse CD8a, (clone EPR21769)	Abcam	Cat: ab217344, RRID: AB_2890649
Anti-mouse/human GL7, FITC (clone GL7)	Biolegend	Cat: 144603; RRID: AB_2561696
Anti-mouse Cxcr4, PerCP-CY5.5 (clone L276F12)	Biolegend	Cat: 146509; RRID: AB_2562786
Anti-mouse FCRL5, APC (Polyclonal)	R&D Systems	Cat: FAB6757A; RRID: AB_3492093
Anti-mouse CCR6, PE-Cyanine7 (clone 29-2L17)	Biolegend	Cat: 129815; RRID: AB_1877244
Anti-mouse CD3e, FITC (clone 17A2)	Biolegend	Cat: 100204; RRID: AB_312661
Anti-mouse CD4, BV711 (clone GK1.5)	Biolegend	Cat: 100447; RRID: AB_2564586
Anti-mouse CD8a, PE/Dazzle 594 (clone 53-6.7)	Biolegend	Cat: 100761; RRID: AB_2564026
Anti-mouse FAS, PE-CF594 (clone Jo2)	BD Biosciences	Cat: 562499; RRID: AB_11154214
Anti-mouse IFNy, BV650 (clone XMG1.2)	Biolegend	Cat: 505832; RRID: AB_2734492
Anti-mouse Perforin 1, APC (clone eBioOMAK-D)	Invitrogen	Cat:17-9392-80 RRID: AB_469514
Anti-mouse Granzyme B, PE-Cyanine7 (clone NGZB)	Invitrogen	Cat: 25-8898-82; RRID: AB_10853339
Anti-mouse CD20, (clone GOT214A)	Abcam	Cat: ab271288; RRID: AB_3451983
Anti-mouse CD8a, (clone C8/144B)	Abcam	Cat: ab17147; RRID: AB_443686
Anti-mouse Ki67, (clone B56)	Abcam	Cat: ab279653; RRID: AB_2934265
Anti-mouse/human Phospho-Zap-70, (clone 65E4)	Cell Signaling Technology	Cat: 2717; RRID: AB_2218658
Anti-mouse/human Syk+Zap70, (clone EPR26232-39)	Abcam	Cat: ab300410; RRID: AB_3716881
Anti-mouse CD8a Alexa Fluor 647antibody (clone EPR21769)	Abcam	Cat: ab237365; RRID: AB_3716882
Anti-mouse F4/80, (Clone CI:A3-1)	Abcam	Cat: ab6640; RRID: AB_1140040
Goat F(ab) anti-mouse IgG H&L	Abcam	Cat: ab6668; RRID: AB_955960
Rabbit (DA1E) mAb IgG Isotype Control	Cell Signaling Technology	Cat: 3900; RRID: AB_1550038
Goat anti-rat IgG H&L, Alexa Fluor 568	Abcam	Cat: ab175476; RRID: AB_2813739
Goat anti-mouse IgG H&L, Alex Fluor 488	Abcam	Cat: ab150113; RRID: AB_2576208
Goat anti-rabbit IgG H&L, Alexa Fluor 488	Abcam	Cat: ab150077; RRID: AB_2630356
Goat anti-rabbit IgG H&L, Alexa Fluor 647	Abcam	Cat: ab150083; RRID: AB_2714032
Donkey anti-rat IgG H&L, Alexa Fluor 647	Abcam	Cat: ab150155; RRID: AB_2813835
Goat anti-rabbit IgG H&L, Alexa Fluor 568	Abcam	Cat: ab175471; RRID: AB_2576207
Goat anti-mouse IgG H&L, Alexa Fluor 647	Abcam	Cat: ab150115; RRID: AB_2687948
Goat anti-rat IgG H&L, Alexa Fluor 488	Abcam	Cat: ab150165; RRID: AB_2650997

Biological samples		

2225L Tumor Transplant Line; 2224L Tumor Transplant Line	Tp53^-/-^ Tumors (Jerry et al., 2000, Herschkowitz et al.^66^); Hollern et al.^13,64^	NA

Chemicals, peptides, and recombinant proteins		

Fixable Near-IR Dead Cell Stain, 633 or 635 nm excitation	Invitrogen	Cat: 50-112-1531
LIVE/DEAD^™^ Fixable Aqua Dead Cell Stain, 405 nm excitation	Invitrogen	Cat: L34965
CellTrace^™^ CFSE Cell Proliferation Kit	Invitrogen	Cat: C34570
IHC Antigen retrieval solution, 10x High pH	Invitrogen	Cat: 00-4956-58
Fingolimod (FTY720)	Caymen Chemical	Cat: 10006292
Incucyte^®^ Cytolight Rapid Dye	Sartorius	Cat: 4706
Incucyte^®^ Annexin V Dye	Sartorius	Cat: 4642
Recombinant Mouse IL-4	Biolegend	Cat: 574306
Mouse BAFF/TNFSF13B/CD257 Protein	ACROBiosystems	Cat: BAF-M521Y
Mouse IL-2 Recombinant Protein	Peprotech	Cat: 212-12
Mouse IL-7 Recombinant Protein	Peprotech	Cat: 217-17

Critical commercial assays		

Chromium Single Cell 3' Reagent Kits	10x Genomics	Chromium^™^Single Cell 3' Library & Gel Bead Kit v2, PN-120237; Single Cell 3' Chip Kit v2 PN-120236; i7 Multiplex Kit PN-120262
Chromium Single Cell 5' Reagent Kits	10x Genomics	Chromium Next GEM Single Cell 5' Kit v2, PN-1000265; Library Construction Kit, PN-1000190; Chromium Next GEM Chip K Single Cell Kit, PN-1000287; Dual Index Kit TT Set A, PN-1000215
MACS Tumor Dissociation Kit, mouse	Miltenyi	Cat: 130-096-730
Pan B Cell Isolation Kit II mouse	Miltenyi	Cat: 130-104-443
CD8a T cell Isolation Kit	Miltenyi	Cat: 130-104-075
Qiagen RNeasy Mini Kit	Qiagen	Cat: 74104
TruSeq Stranded mRNA Library Prep kit	Illumina	Cat: 20020595
Mouse CXCL9/MIG DuoSet ELISA	R&D Systems	Cat: DY492-05

Deposited data		

Raw data; RNA-seq	Sikov et al.^70^	CALG40603
Raw and normalized data; microarray	Miyake et al.^68^	GSE32646
Raw data; RNA-seq	Echavarria et al.^69^	https://doi.org/10.1158/1078-0432.CCR-17-1912
Raw data and normalized data; RNA-seq	Hollern et al.^13^	GSE124821
Raw and normalized data; scRNA-seq	Hollern et al.^13^	GSE136206
Raw data; RNA-seq	This Paper	GSE276004
Raw data; scRNA-seq	This Paper	GSE276006

Experimental models: Cell lines		

KPB25Luv	Hollern et al.^13,64^	NA
T11-Apobec	Hollern et al.^13,64^	NA

Experimental models: Organisms/strains		

Mouse: FVB/NJ	The Jackson Laboratory	JAX: 001800
Mouse: Balb/cJ	The Jackson Laboratory	JAX: 000651

Software and algorithms		

Seurat (version 5.0.0)	Satija et al.^139^; Hao et al.^140^	https://github.com/satijalab/seurat
CellRanger (version 3.0.2)	10x Genomics	https://support.10xgenomics.com/single-cell-gene-expression/software/pipelines/latest/what-is-cell-ranger
CellRanger (version 6.1.1)	10x Genomics	https://support.10xgenomics.com/single-cell-gene-expression/software/pipelines/latest/what-is-cell-ranger
Cellchat (version 2.1.0)	Jin et al.^103^	https://github.com/sqjin/CellChat
R (version 4.3.2)	R Core Team	https://www.R-project.org/
R studio	R Studio	https://www.rstudio.com
Star aligner	Dobin et al.^141^	https://github.com/alexdobin/STAR
SALMON	Patro et al.^142^	https://combine-lab.github.io/salmon/
Picard	Broad Institute	https://broadinstitute.github.io/picard/
Cluster 3.0	de Hoon et al.^143^	http://bonsai.hgc.jp/~mdehoon/software/cluster/software.htm
Java TreeView	NA	http://bonsai.hgc.jp/~mdehoon/software/cluster/software.htm
SAMtools	Li et al.,^144^	http://www.htslib.org
SigClust	Huang et al.^145^	https://cran.r-project.org/web/packages/sigclust/index.html
FlowJo (version 10.10)	FlowJo, LLC	https://www.flowjo.com/solutions/flowjo/downloads/
GraphPad Prism	GraphPad Software	https://www.graphpad.com/
QuPath (version 0.5.0)	Bankhead et al.^146^	https://github.com/qupath/qupath

Other		

Matrigel^®^ Basement Membrane Matrix	Corning	Cat: 354234
Nunctrade MaxiSorptrade ELISA Plates, Uncoated	Biolegend	Cat: 423501
Millicell 3UM Pet Hang, 24Well	Sigma-Aldrich	Cat: PTSP24H48
HUMEC READY MEDIUM-(With supplement)	Life Technologies Inc.	Cat:12752010
RPMI 1640	Life Technologies Inc.	Cat: 11875093

## Highlights

- CD40-activated B cells drive anti-tumor immunity and CD8^+^ T cell tumor infiltration
- CD40-activated B cell interactions with macrophages can elicit CD8^+^ T cell migration
- CD40-activated B cells organize aggregates containing CD8^+^ T cells
- CD40-activated B cells interact with CD8+T cells and elicit CD8^+^ T cell cytotoxicity
