# Supplementary material for: B cell responses to CD40 stimulation ignite anti-tumor immunity by activating CD8+ T cell cytotoxicity
Source: Cell Rep. Author manuscript; Available in PMC 2026 Oct 28. (PMC13716135; doi:10.1016/j.celrep.2026.117957)
Supplement: Supplementary file 1 [file NIHMS2213384-supplement-1.pdf]

Cell Reports, Volume *45*

Supplemental information

B cell responses to CD40 stimulation ignite anti-tumor immunity by activating $CD8^+$ T cell cytotoxicity

Kendrick Nguyen, Monika Quackenbush, Sara Basbous, Haley Rodriguez, Georgia Lattanzi, Elsie Quansah, Chynna Bowman, Sammy Weiser Novak, Daniela Boassa, Jingting Yu, Taylor Ozbun, Asona Lui, Aatish Thennavan, Peter Watson, April Wehner, and Daniel Hollern

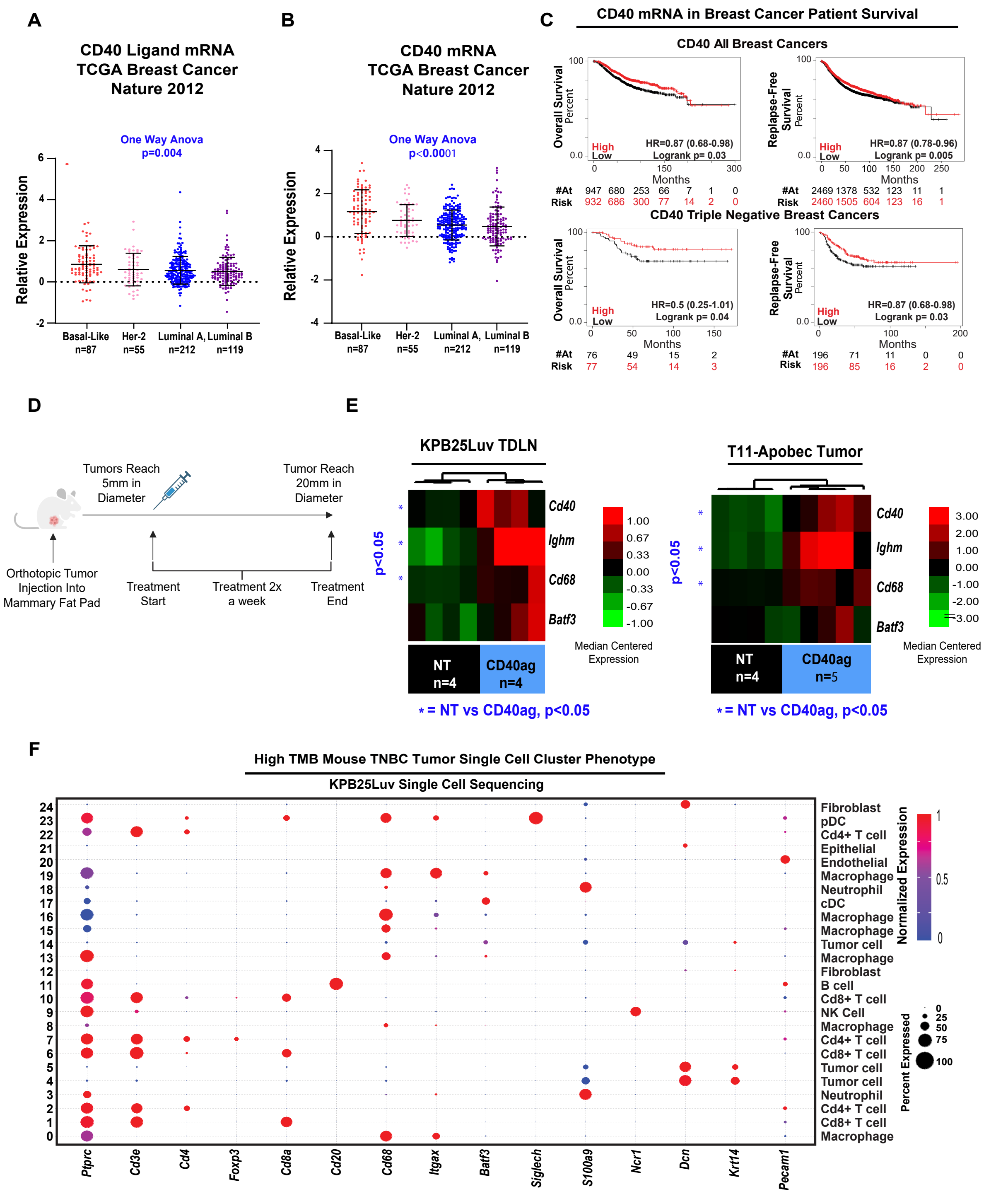

Supplemental Figure 1: CD40 and CD40 ligand activity is frequently elevated in mouse and human TNBCs.
A) CD40lg mRNA expression across tumors from different human breast cancer intrinsic subtypes (Basal-like, Her-2, Luminal A, Luminal B) in publicly available TCGA data (Cancer Genome Atlas Network, 2012).The p-value was derived by a one way ANOVA to detect significant variance between subtypes. B) CD40 mRNA expression from tumors across different human breast cancer intrinsic subtypes (Basal-like, Her-2, Luminal A, Luminal B) in publicly available TCGA data (Cancer Genome Atlas Network, 2012). The p-value was derived by a one way ANOVA to detect significant variance between subtypes. C) Overall survival and relapse free survival data from patients with tumors from all breast cancer subtype and also TNBC. Results depict the relationship between CD40 and survival. Dataset is publicly available at KMplot.com. The p-value were derived by Kaplan Meier analysis. D) Cartoon model depicting how in vivo survival studies are conducted. Mouse TNBC cells are injected orthotopically into the mouse mammary fat pad. Tumors are allowed to reach 5mm in diameter. Once they reach 5mm in diameter antibody treatment is given 2x a week until tumors reach 20mm in diameter. This is considered end stage tumors, and the mice are euthanized according to IACUC protocol. E) Heatmap of bulk mRNA expression of CD40, Ighm, Cd68, and Batf3 of nontreated or CD40ag treated KPB25Luv TDLNs along with T11 Apobec Tumors. p-Value is calculated by DESEQ2 analysis and is adjusted for false discovery rate. F) Dot plot of mRNAs used for baseline cell phenotyping and individual marker analysis of KPB25Luv single cell sequencing.

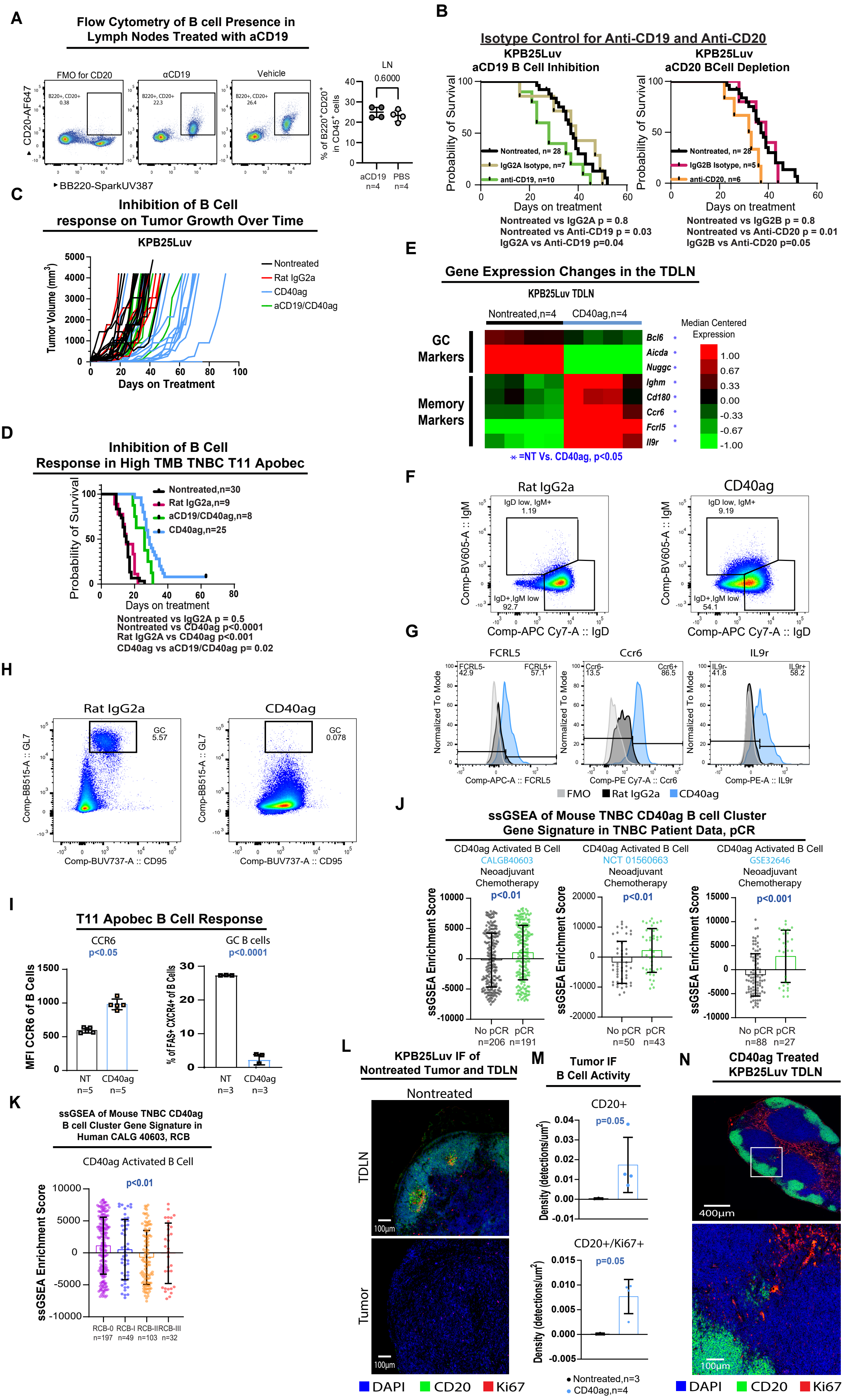

Supplemental Figure 2: CD40 agonist drives a non-germinal center B cell response in mouse TNBCs. A) Flow cytometry of B220+ CD20+ cells of T11 Apobec lymph nodes treated with anti-CD19. B) Survival of KPB25Luv TNBC mouse model treated with Rat IgG2A isotype control, Rat IgG2B isotype control, anti-CD19, or anti-CD20. P-value was calculated by Kaplan Meier analysis comparing CD40 agonist treated versus nontreated. NS= not significant. C) Tumor growth over time of KPB25Luv TNBC mouse model treated with Rat IgG2A isotype control, CD40ag, or CD40ag/anti-CD19. D) Survival of T11 Apobec TNBC mouse model treated with Rat IgG2A isotype control, CD40ag, or CD40ag/anti-CD19. Depicted comparative p-values were calculated by Kaplan Meier analysis. E) Heatmap of bulk mRNA differentially expressed genes of key germinal center and memory B Cell markers of nontreated or CD40ag KPB25Luv TDLNs. p-Value is calculated by DESEQ2 analysis and is adjusted for false discovery rate. F) Flow plots for flow cytometry cellular quantification of IgD (low) IgM+ B cells gated on B220+ CD19+ from the TDLNs of KPB25Luv bearing mice treated with CD40ag antibody or rat IgG2A isotype control antibody seen in figure 2C. G) Flow plots for MFI quantification of FCRL5, CCR6, IL9r in live B220+ CD19+ IgM+ IgD low B cells from the TDLNs of KPB25Luv bearing mice treated with CD40ag antibody or rat IgG2A isotype control antibody seen in figure 2C. H) Flow plots for flow cytometry quantification of FAS+ GL7+ B cells gated on B220+ CD19+ B cells from TDLNs of KPB25Luv bearing mice treated with CD40ag antibody or rat IgG2A isotype control antibody seen in figure 2D. I) Flow cytometry of CCR6+ B cells and Fas+/Cxcr4+ B cells in T11 Apobec TDLNs. Statistical p-value determined by two-tailed unpaired Mann-Whitney testing. J) Single sample gene set enrichment analysis (ssGSEA) scoring of the scSEQ-derived CD40ag activated B cell gene signature in human datasets CALGB40603, NCT 01560663, and GSE32646. No PCR / PCR relates to pathological complete response. Statistical p-value was determined by a two-tailed unpaired T-test. K) ssGSEA of the scSEQ-derived CD40ag activated B cell gene signature in human datasets CALGB40603. RCB scores relates to residual cancer burden. Statistical p-value determined by one-way ANOVA to detect significant variance between subtypes. L) Immunofluorescence staining for DAPI (blue), CD20 (green), and KI67 (red) in non-treated control KPB25Luv tumors along with TDLNs. Scale bars represent 100µm. M) Qupath immunofluorescence quantification of CD20+ cells and CD20+/Ki67+ Cells from non-treated control or CD40ag treated KPB25Luv tumors. Statistics and p-value are calculated by a two-tailed unpaired Mann Whitney test. N) Immunofluorescence staining for DAPI (blue), CD20 (green), and KI67 (red) in CD40 agonist treated KPB25Luv tumors along TDLNs. Squared are B cells outside the follicle. Scale bars represents 400µm (top) and 100µm (bottom).

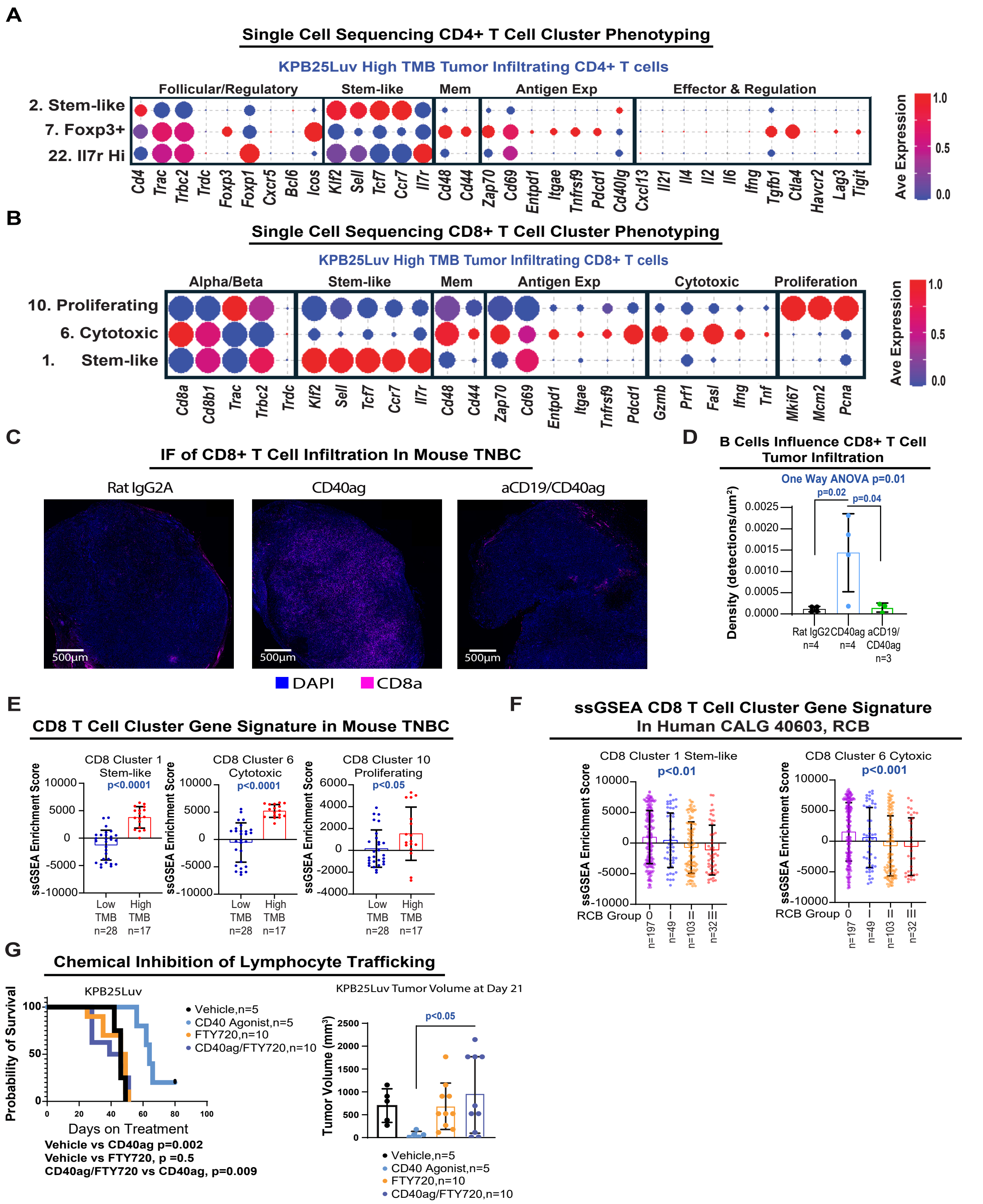

Supplemental Figure 3: Characterization of T cell responses to KPB25Luv tumors and CD40ag. A) Dot plot of genes used to phenotype CD4+ T cell clusters in the KPB25Luv scRNA-seq UMAP. B) Dot plot of genes used to phenotype CD8+ T cell clusters in the KPB25Luv scRNA-seq UMAP. C) Immunofluorescence staining for DAPI (blue) and CD8a (magenta, CD8+ T cells) in Rat IgG2A isotype control treated tumors, CD40ag treated tumors, and CD40ag with anti-CD19 B cell inhibition treated tumors. Scale bars represent 500µm. D) Qupath quantification of CD8a (CD8+ T cells) density in Rat IgG2A isotype control treated tumors, CD40ag treated tumors, and CD40ag with anti-CD19 B cell inhibition treated tumors. Statistics represent a one way ANOVA test with Tukey's multiple comparisons. E) Single sample gene set enrichment analysis (ssGSEA) of scSEQ KPB25Luv tumors derived signatures of stem-like CD8 T cells, cytotoxic CD8 T cells, and proliferating CD8 T cells in published bulk RNA-sequencing dataset (GSE124821) of primary tumors from low TMB (n=28) and high TMB (n=17) TNBC mouse models (See PMID: 31730857). F) Single sample gene set enrichment analysis (ssGSEA) scoring of KPB25Luv tumors derived signatures of stem-like CD8 T cells, cytotoxic CD8 T cells, and proliferating CD8 T cells human dataset CALGB40603 RCB scores relates to residual cancer burden. Statistical p-value determined by one-way ANOVA. RCB0 (n=197), RCBI (n=49), RCBII (n=103), RCBIII(n=32). G) Mouse survival and tumor volume of KPB25Luv TNBC mouse model treated with vehicle, CD40ag, FTY720, or CD40ag/FTY720 at 21 days. Depicted compararive p-values were calculated by Kaplan Meier analysis. Right, measurement of KPB25Luv during the acute response timepoint of 21 days treated with vehicle, CD40ag, FTY720, or CD40ag/FTY720. Statistics and p-value are calculated by a two-tailed unpaired Mann Whitney test.

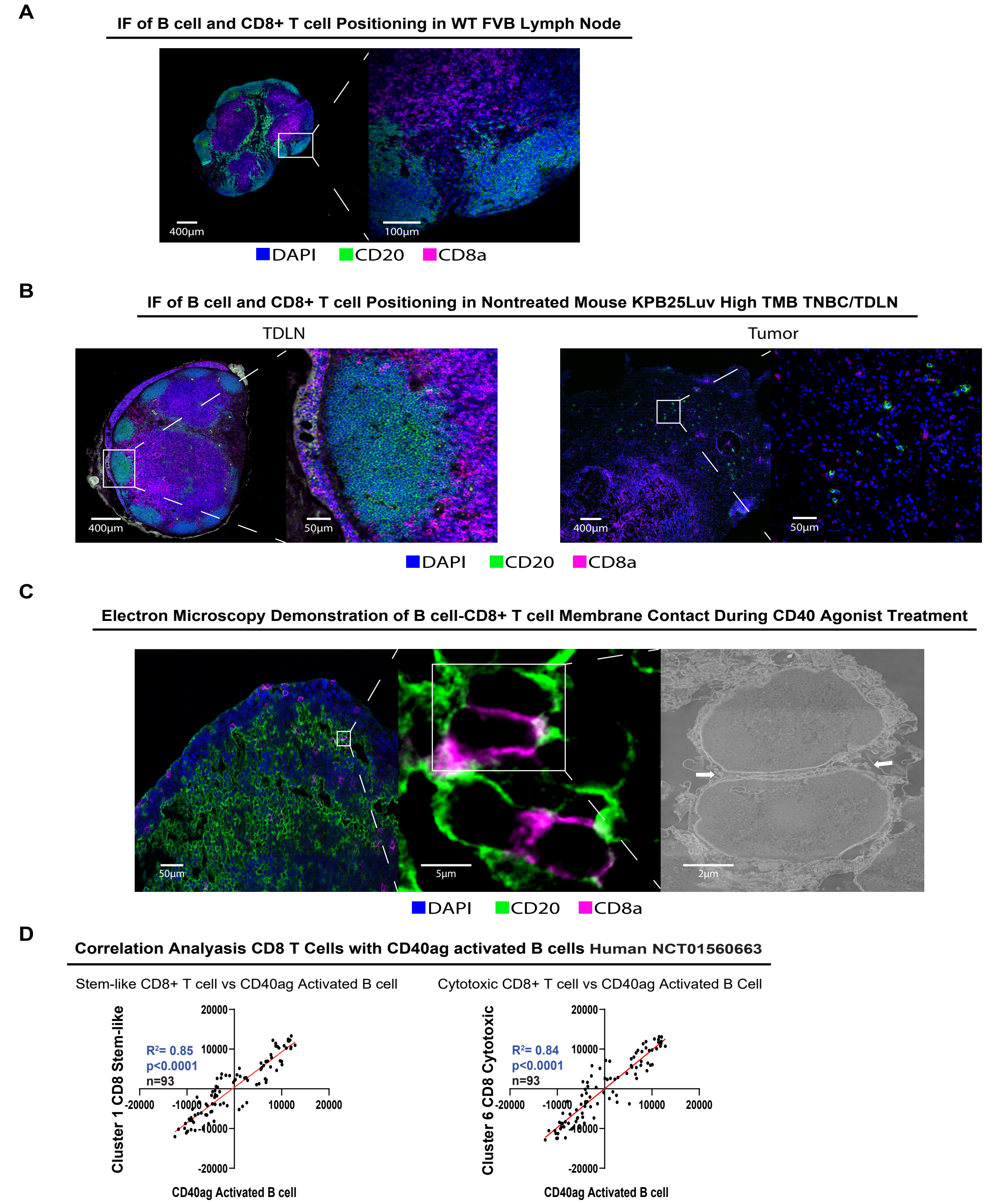

Supplemental Figure 4: Characterization of B cell – CD8+ T cell interactions in in tumor naïve lymph nodes, nontreated tumor draining lymph nodes and tumors, CD40ag treated tumor draining lymph nodes, and human clinical trial RNA-seq data.

A) Immunofluorescence staining for DAPI (blue), CD20 (green), and CD8a (magenta) in a tumor free FVB mouse and the axillary lymph node. Scale bars from left to right represents 400µm (zoomed out) and 100µm (zoomed in). B) Immunofluorescence staining for DAPI (blue), CD20 (green), and CD8a (magenta) in nontreated KPB25Luv tumors along with TDLNs. Scale bars for TDLN from left to right represents 400µm (zoomed out) and 50µm (zoomed in). Scale bars for tumors from left to right represents 400µm (zoomed out) and 50µm (zoomed in). C) Correlative electron and light microscopy (CLEM) of CD40ag treated KPB25Luv TDLNs focused on the cellular interface between a CD20+ cell and CD8+ cell in a B cell follicle. Scale bars from left to right represents 50µm, 5µm, and 2µm. D) Correlation plot of either stem-like CD8 T cell or cytotoxic CD8 T cell gene signature with the mouse CD40ag activated B cell gene signature in human dataset NCT 01560663. R-squared and p-value calculated by linear regression analysis in graphpad prism. All signatures derived from KPB25Luv tumor single cell sequencing, using top 100 genes (removing those linked to proliferation). Data was scored by single sample gene set enrichment analysis (ssGSEA).

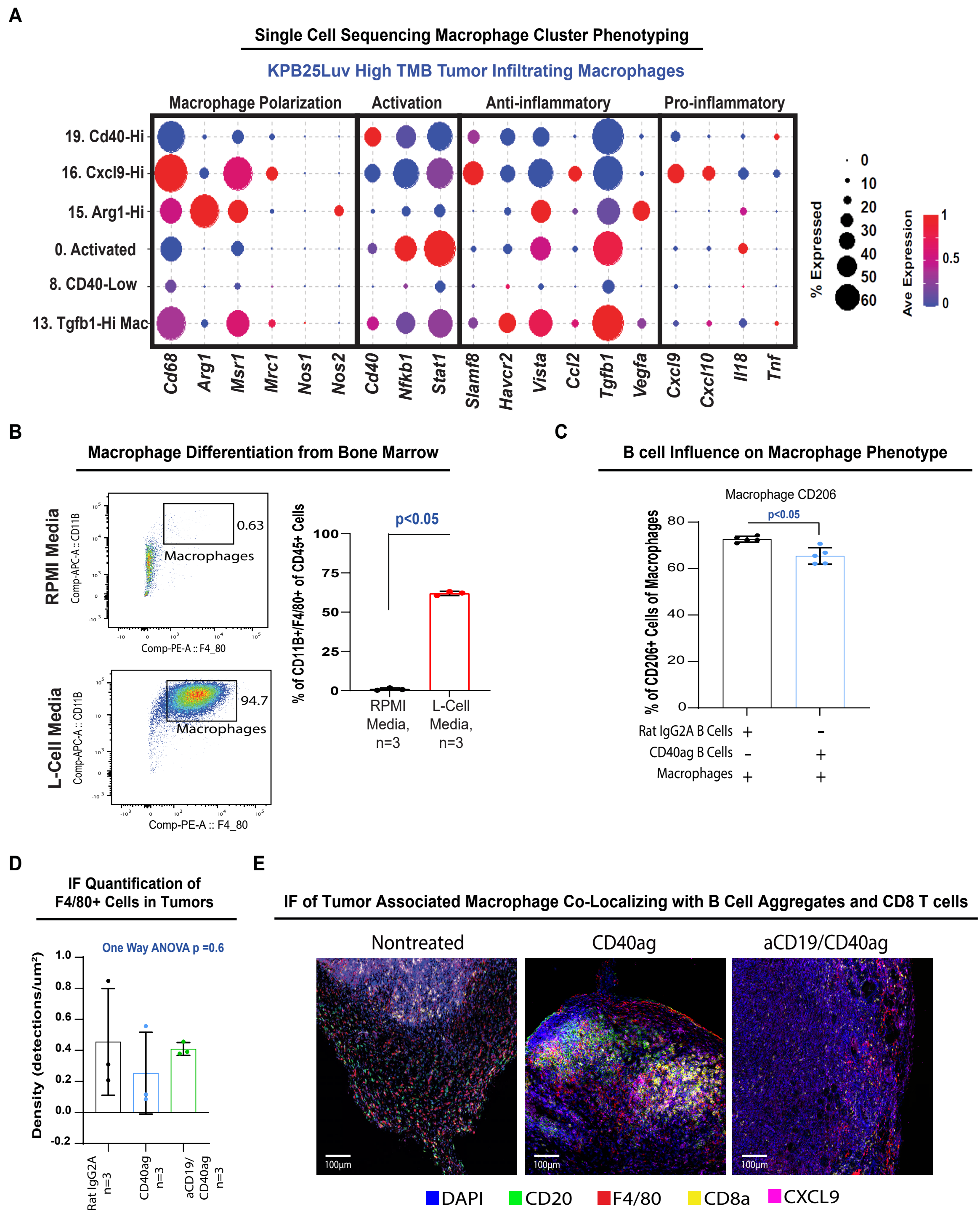

Supplemental Figure 5: CD40 agonist driven B cell activation impacts macrophage phenotypes and interactions with CD8+ T cells.
A) Dot plot of genes used to identify macrophage phenotypes across clusters in the integrated UMAP. B) Flow cytometry of CD11b+ F4/80+ cells from BMDM differentiation using L-cell media or RPMI media. C) Flow cytometry of CD206 expression in BMDMs cocultured with Rat IgG2A isotype control or CD40ag treated B cells isolated from KPB25Luv TDLNs. D) Qupath immunofluorescence quantification of F4/80+ cell density across KPB25Luv tumors treated with Rat IgG2A isotype control, CD40ag, or aCD19/CD40ag. Statistical p-value derived from a one way ANOVA. E) Immunofluorescence for DAPI (blue), F4/80 (red), CXCL9 (magenta), CD8a (yellow), and CD20 (green) in a non-treated control tumor, CD40ag treated tumor, and a aCD19/CD40ag treated KPB25Luv tumor. Scale bars represent 100µm.

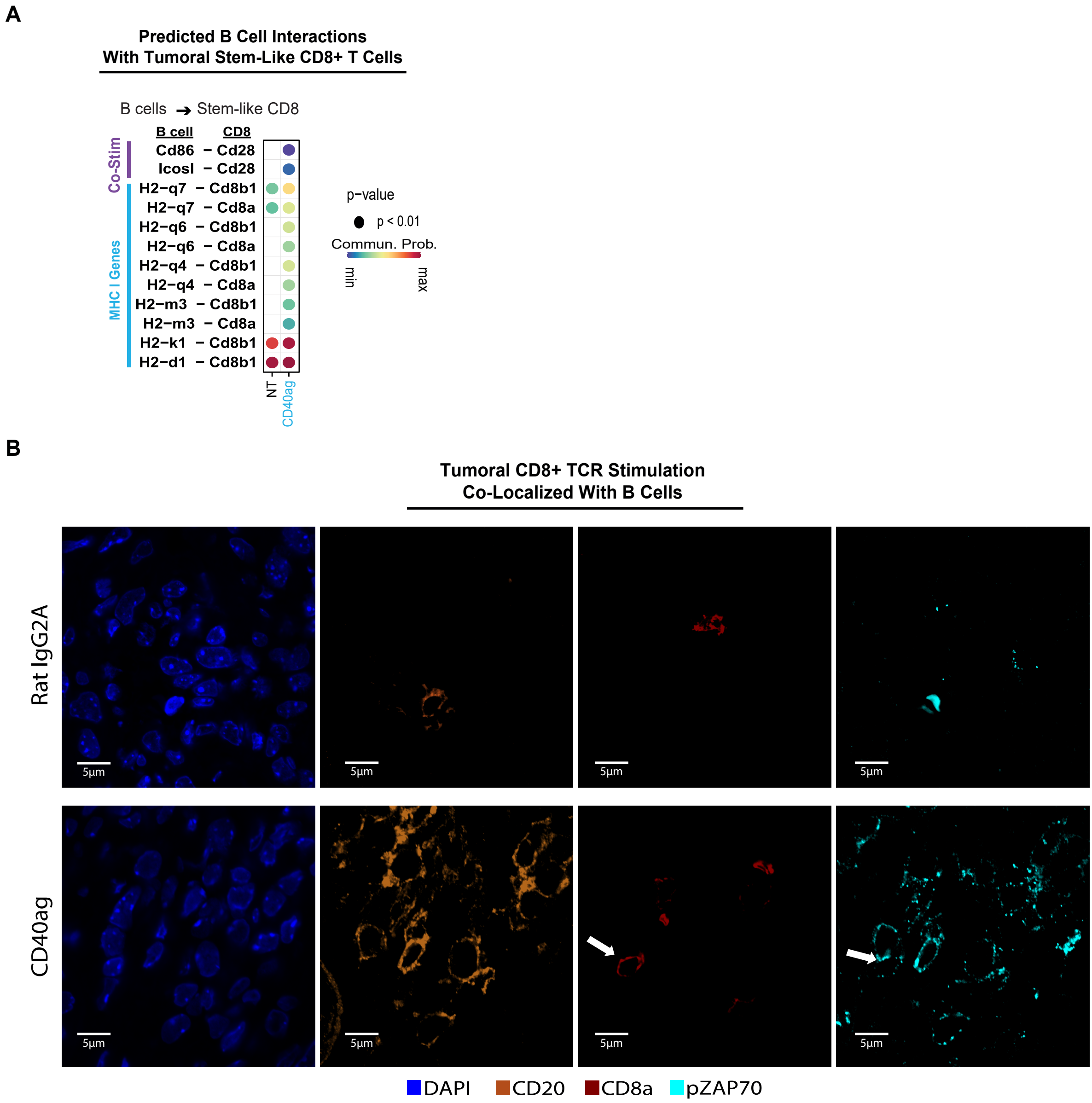

Supplemental Figure 6: B cell Responses to KPB25Luv tumors and CD40ag Activate CD8+ T cells. A) Cellchat dot plot of key B cell interactions with stem-like CD8+ T cells within CD40ag treated and nontreated KPB25Luv tumors. The dot color and size is indicative of the calculated communication probability and p-values which are respectively computed from a one-sided permutation test. B) Immunofluorescence of DAPI (blue), CD8 (red), phosphorylated Zap70 (pZap70) (cyan), and CD20 (orange) from Rat IgG2A isotype control and CD40 agonist treated KPB25Luv tumors. Scale bars represent 5μm.
